# Trawling the depths of gene regulation: transcription–factor families orchestrating immunity in teleost fish

**DOI:** 10.3389/fimmu.2026.1938274

**Published:** 2026-10-01

**Authors:** Alexander Rebl, Bertrand Collet, Jiří Kyslík, Tomáš Korytář

**Affiliations:** 1 Research Institute for Farm Animal Biology (FBN), Dummerstorf, Germany; 2 Université Paris-Saclay, INRAE, UVSQ, VIM, Jouy-en-Josas, France; 3 Institute of Parasitology, Biology Centre Czech Academy of Science, České Budějovice, Czechia; 4 Institute of Aquaculture and Protection of Waters, South Bohemian Research Centre of Aquaculture and Biodiversity of Hydrocenoses, Faculty of Fisheries and Protection of Waters, University of South Bohemia in Ceske Budejovice, České Budějovice, Czechia

**Keywords:** aquatic health, cytokines, fish immunity, functional genomics, gene regulatory networks, immune regulation

## Abstract

Transcription factors act as central regulators of immune responses by integrating signals from immunological pathways and translating them into cell type-specific gene-expression programmes. Dynamic networks of transcription factors and co-factors control the activation, polarisation and functional specialisation of immune cells, thereby coordinating the magnitude, timing and specificity of responses to pathogenic stimuli whilst promoting the restoration of homeostasis. Although immune-relevant transcription factors have been extensively studied in mammals, the molecular principles governing transcriptional initiation and regulation by transcription factor networks remain incompletely understood across vertebrates. This knowledge gap is particularly evident in teleost fish, which possess unique anatomical and genomic features, including lineage-specific gene duplications that have shaped immune regulatory networks. Moreover, teleosts have evolved or expanded immune-cell functions that may involve novel combinations of conserved transcription factors, potentially giving rise to regulatory modules distinct from those characterised in mammals. It remains unclear how defined immunological stimuli are translated into stimulus-specific transcriptional programmes through the coordinated action of signalling pathways, transcription factors and chromatin-associated regulatory mechanisms. This review focuses on the transcriptional regulation of immune responses in teleost fish and provides an overview of immune-relevant transcription factors involved in immediate-early and delayed primary responses, as well as secondary and lineage-specific transcriptional programmes in representative model–fish species. By integrating current knowledge from teleost models, this review aims to establish a framework for understanding immune transcriptional regulation in fish, highlight conserved and lineage-specific regulatory mechanisms and identify key challenges for future research in non-mammalian vertebrate immunology.

## 1 Introduction

Immune defence mechanisms have existed for over a billion years (1). Even simple organisms like sponges (2) or cnidarians (3) possess immune systems capable of distinguishing ‘self’ from ‘nonself’ to allow mounting protective responses against infection. With an estimated 30,000 (4) or more species, bony fish exhibit immune systems that have evolved in tandem with their ecological niches and the unique pathogen landscapes they encounter (5). The understanding of teleost immune function is, however, largely limited to a few model species (6–8)—and even in those, it remains incomplete.

As with all vertebrates, it is still unclear how the immune system is precisely regulated across its various phases and under differing physiological demands. This is largely due to the complexity of coordinating innate and adaptive responses, modulating signalling pathways and tailoring immune activity to the nature of the pathogen, the affected host tissue and the organism’s developmental or environmental context. Underlying these advanced immune systems, proven effective across evolutionary timescales, is a complex network of interacting factors that regulate immune-gene expression by binding to specific DNA sequences. These elements operate through finely tuned mechanisms to coordinate precise timing and magnitude of immune activation, the resolution of inflammation and the prevention of excessive tissue damage. This review examines current knowledge of immune-gene regulation in teleost fish, with particular focus on those factors that induce the activation of preformed immune effectors as well as immune gene expression to initiate signalling cascades in specialised immune cells.

### 1.1 Pathogen detected–what next?

Effective immune defence begins with pathogen detection. Conserved microbial components such as bacterial lipopolysaccharides (LPS), viral RNA or fungal zymosan are known as pathogen-associated molecular patterns (PAMPs). Once they bind to pattern recognition receptors (PRRs) (9), they activate signalling cascades that coordinate pathogen defence and immune cell activation (10, 11).

Vertebrate genomes encode a diverse set of PRRs, each attuned to a specific set of ligands. Amongst them, toll-like receptors (TLRs) represent the most studied PRR type (12, 13). In addition, membrane-bound C-type lectins (CLRs) as well as intracellular receptors such as NOD-like receptors (NLRs) and cytosolic DNA sensors play key roles in detecting pathogens and triggering immune responses (14). Many pathogens are recognised by multiple PRRs simultaneously, often converging on shared adaptor proteins and overlapping signalling pathways. In this way, distinct PAMPs derived from the same pathogen can activate overlapping, yet partially divergent, sets of immune transcription factors. Co-infecting pathogens, resident microbiota, host genetic background and the cellular microenvironment further complicate the attribution of signalling responses to specific stimuli.

Despite this bewildering complexity bearing both redundant and alternative signalling, these interconnected pathways converge on the activation of distinct (sets of) transcription factors, which ultimately shape the immune response at the transcriptomic level by manipulating the concentration of gene transcripts that become translated into proteins. In other words: transcription factors guide the way the same genetic blueprint in every host cell is interpreted to generate a suitable physiological response to environmental stimuli.

## 2 Hooked on immune genes: the three levels of transcription-factor activity

When nuclein was first isolated from white-blood cell nuclei in the 1860s, scientists debated whether its protein fraction or the phosphate-rich substance later named deoxyribonucleic acid (DNA) carried hereditary information. More than a century and a half later, it is well established that DNA does contain about 20,000 protein-coding genes, which define the structure and characteristics of mammalian individuals (15). Bony fishes have a comparable number of genes in compact genomes such as that of the pufferfish (*Takifugu rubripes*), whereas fish species that underwent additional whole-genome duplications, like salmonids or cyprinids (16, 17), probably carry almost twice as many genes. DNA-associated proteins, however, play equally crucial roles. They not only package the genome but also orchestrate when and how the genetic script is read: histones shape DNA accessibility across nearly the entire genome, whilst transcription factors attach to particular DNA sequences to selectively activate or suppress genes.

In textbooks and countless research articles, the ‘transcription story’ is usually depicted as a straightforward mechanism. Usually, a single specific stimulus triggers a well-orchestrated cascade of protein interactions, culminating in a defined response—or even a spectrum of finely tuned reactions. In reality, these orderly cascades are built on fleeting, nanosecond-long interactions amongst distinct biopolymers with complementary chemobiological affinities, occurring within the crowded milieu of micro- and macromolecules. At their core, all specificity arises from the precise fit of a transcription factor into the unique three-dimensional structure of DNA (18, 19) and from the physical contact between the factor’s amino acid side chains and the atoms of the DNA-base pairs. These contacts span on average 24 amino acid residues and 12 nucleotides (20) and include not only hydrogen bonds but also van der Waals forces and electrostatic interactions (21). Through this combination of structural complementarity and multiple weak forces, transcription factors can achieve a binding preference for their cognate response elements that may exceed 1,000-fold compared with non-specific DNA sequences (22).

RNA polymerase II is the vital enzyme producing the transcript of the genomic instructions for the protein-making machinery. Basal transcription factors (TFIID, -IIB, -IIF, -IIE, -IIH, -IIA) are necessary for this RNA polymerase II-mediated transcription process of essentially all vertebrate protein-coding genes (23). Unlike basal factors, specific transcription factors bind cognate DNA response elements (24, 25) to induce or repress target genes by modulating DNA accessibility or RNA polymerase recruitment (26). Approximately 7% to 8% of all protein-coding sequences in multicellular organisms are attributed to transcription factors (27). Their ability to regulate gene expression depends on conserved DNA-binding domains, including zinc fingers, helix-turn-helix (including homeodomains), helix-loop-helix, helix-span-helix or leucine zipper domains, which mediate sequence-specific DNA recognition (28). The recognition of a matching DNA sequence typically occurs through a DNA-binding domain that positions the factor near its target. Transcription factors also regulate gene expression through interactions with cofactors, either coactivators or corepressors, or by being recruited via signalling pathways triggered by hormones or growth factors.

Research over the past three decades has documented that teleost immune responses are governed by the same fundamental principles as those of other vertebrates (29–31). What makes transcriptional regulation particularly interesting in teleosts is the evolutionary history of their genomes. The ancestral teleost genome duplication and subsequent lineage-specific duplication events (16, 17) generated expansions of numerous gene families, some of which have retained multiple immune-relevant paralogues, providing substrates for subfunctionalisation and regulatory diversification. In addition, the anatomical context of teleost transcription-factor programmes differs significantly from that of mammals. The absence of bone marrow and lymph nodes, together with the prominent roles of the head kidney, spleen and mucosal tissues, means that immune-cell development, activation and effector functions are distributed across anatomical compartments that differ substantially in their organisation and functional roles from those in mammals.

Ruslan Medzhitov and Tiffany Horng introduced a simplified view of transcriptional immune gene regulation, in which the large diversity of transcription factors is organised into three functional classes (32). This classification provides a useful framework for discussing teleost transcription-factor networks (**Figure 1**) whilst recognising that these three levels are functionally interconnected rather than strictly sequential. The following sections therefore focus on representative transcription-factor families in teleost fish, with particular emphasis on their evolutionary diversification, paralogue retention, regulatory interactions and experimentally demonstrated roles in immune responses.

**Figure 1:**
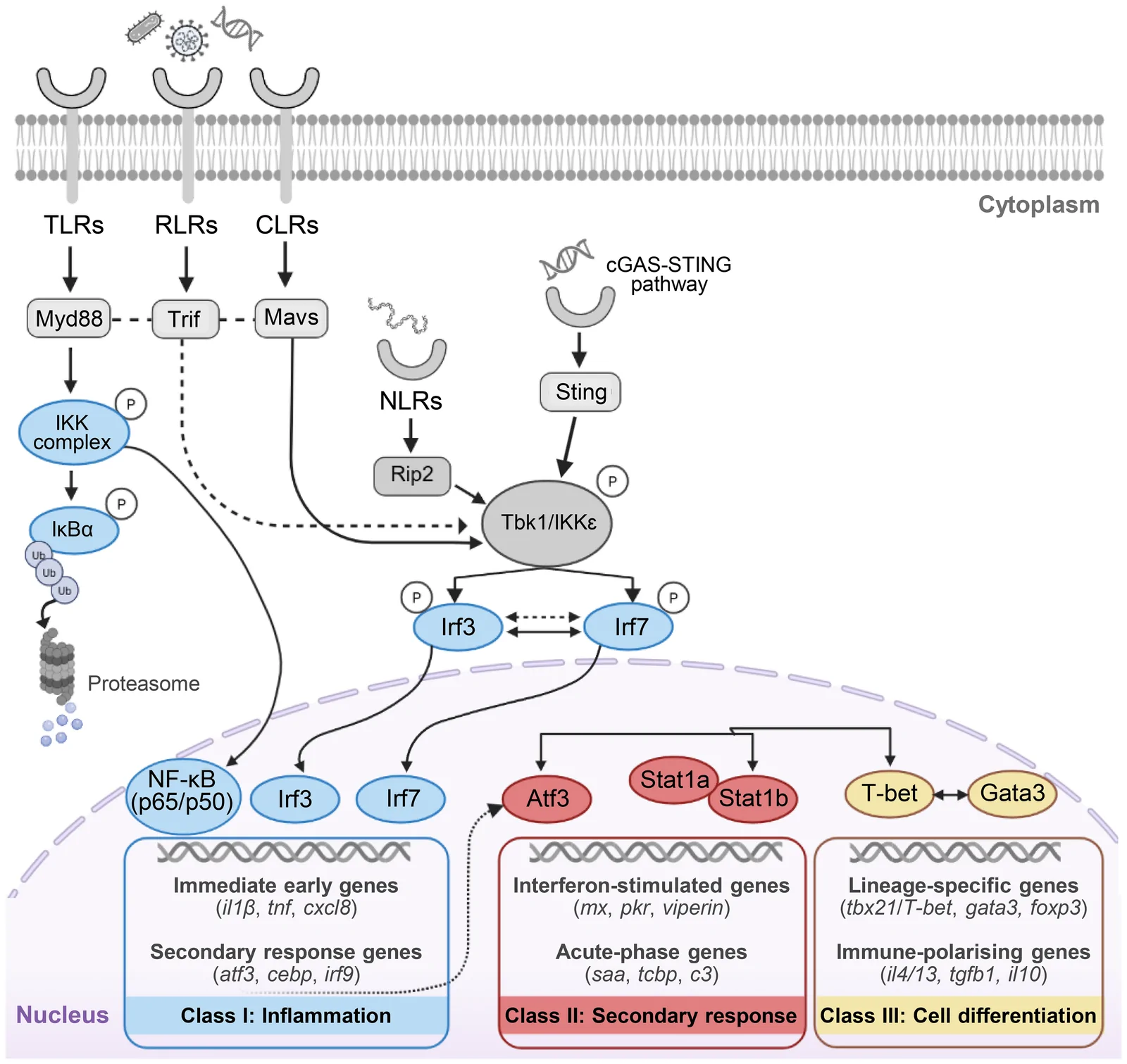
The three classes of transcription factors. This simplified scheme highlights the integration and interaction of transcription-factor pathways underlying coordinated immune responses in teleosts. Signals from pattern-recognition receptors, including TLRs, RIG-I-like receptors (RLRs), CLRs, NLRs and the cGAS–STING pathway, converge on primary transcription factors (class I, inflammation), such as NF-κB and Irf3/7. They induce the expression of secondary transcription factors (class II, secondary responses), including Stat1 and Atf3. Together with lineage-determining factors (class III, cell differentiation), including T-bet and Gata3, these transcription factors form interconnected regulatory modules that coordinate distinct immune-gene programmes. Only representative transcription factors from each class are shown as examples of a larger set of factors. Created with BioRender.com.

### 2.1 Level I: primary transcription factors for immediate early and delayed primary immune responses

Class-I transcription factors do not require *de novo* protein synthesis and are activated immediately downstream of PRR-mediated signalling (**Figure 1**). They are constitutively expressed and typically reside in the cytoplasm, from where they can translocate into the nucleus, often within half an hour after stimulation (33). Once inside the nucleus, these ‘primary transcription factors’ induce the expression of primary response genes including the so-called ‘immediate-early genes’ (IEGs) (**Figure 1**). IEG transcripts and proteins generally exhibit short half-lives (34), consistent with their transient functional role, as IEG products can be rapidly degraded by the proteasome, even in the absence of ubiquitination (35). Pathogens, in turn, exploit similarly disruptive mechanisms to suppress IEG activity during early stages of infection (36). This is counterproductive for the host, as class-I transcription factors initiate inflammation and set up the regulatory cascade by inducing pro-inflammatory cytokines such as interleukin-1 beta (IL1B) and tumour necrosis factor (TNF), as well as class-II transcription factors (see Section 2.2) that drive the subsequent expression of a broader array of cytokines and immune effectors. In addition, they are also essential for enabling and shaping first-time antigen recognition by lymphocytic cells of the adaptive immunity (37, 38).

At slightly later time points, ‘delayed primary response genes’ (DPRG) are induced (39, 40). This delay is intrinsic and arises from regulatory and structural features of the genes themselves, rather than from a time lag due to newly synthesised transcription factors. Nevertheless, the delayed kinetics are often dependent on newly synthesised cofactors. In addition, DPRGs typically exhibit distinct genomic architectures, including longer transcription units and a higher number of introns, which can complicate and slow down the transcription process. DPRGs are not primed for instant responses but instead represent a key intermediate layer in the cellular response to stimulation, fulfilling functions that differ from those of immediate-early gene (IEG) products. Accordingly, they commonly encode metabolic enzymes, structural proteins and signalling components (39).

Promoters of IEGs often contain composite regulatory elements with closely spaced or overlapping transcription factor–binding motifs, including both proximal and distal enhancer elements, which can assemble into higher-order regulatory complexes known as enhanceosomes, thereby enabling tight and stimulus-responsive transcriptional control (41, 42). Positive feedback loops can sustain nuclear retention and drive secondary waves of gene induction. The activity of class-I transcription factors, however, is usually under tight control: numerous, often redundant mechanisms ensure their rapid inactivation (40) maintaining transcriptional precision to balance pathogen clearance with minimal host damage. Interactions with co-activators and co-repressors further modulate DNA binding and transcriptional activity. IEG transcripts are also frequently regulated by microRNAs and other non-coding RNA species in both mammals (43) and fish (44).

Although the repertoire of transcriptional elements governing fish immunity remains incompletely characterised, decades of research have identified members of the NF-κB/Rel and interferon regulatory factor (Irf) families as key regulators of early immune responses (**Figure 1**). In mammals, NF-κB, together with its co-activator cAMP-response element-binding protein (CREB), initiates inflammatory gene expression, whereas nuclear factors of activated T cells (NFAT), IRF3 and IRF7 primarily regulate calcium-dependent and antiviral responses, including type-I interferon (IFN) induction (45–49). Orthologues of these factors have been identified in teleost fish, and their roles in early immune responses will be explored in the following subsections.

#### 2.1.1 The family of NF-κB and Rel factors—master regulators of inflammation

The NF-κB/Rel family of transcription factors is amongst the best-characterised groups of transcription factors and has profoundly shaped current understanding of immune regulation (50, 51). In most vertebrates, this family comprises five members sharing a highly conserved N-terminal Rel homology domain (RHD) (52). The RHD is essential for nuclear transport, dimerisation and DNA binding. Based on their C-terminal domain, the factors are further divided into the NF-κB proteins NFKB1/p50 and NFKB2/p52 and the Rel factors RelA/p65, RelB and c-Rel. Only RelA, RelB and c-Rel possess a C-terminal transactivation domain (53–55) that recruits the transcriptional machinery including the RNA polymerase II complex. NF-κB/Rel factors bind DNA only as dimers and can also form complexes with other transcription factors as well as co-activators or co-repressors, thereby modulating transcriptional activity (56, 57). NF-κB response elements occur in promoters of IEGs as well as genes involved in acute-phase responses, apoptosis, endoplasmic reticulum (ER) stress and chemical stress (58, 59). These interactions activate conserved signalling pathways that regulate lymphocyte activation, proliferation and apoptosis, making NF-κB a central regulator of innate and adaptive immunity (52, 60–64).

The NFKB1 subunit was first identified in mammals (65) and, 10 years later, in the scup (*Stenotomus chrysops*) via electrophoretic mobility shift assays (EMSAs) (66). One of the first functional characterisations of the teleost NF-κB/Rel signalling pathway was performed in embryonic zebrafish (*Danio rerio*) (67). Here, Rela, Rel and Nfkb2 were initially detected by Northern blot and subsequently cloned. Using EMSA, it was shown that LPS induces the binding of NF-κB/Rel factors to their cognate response elements (derived from mammals) and that the activity of these factors can be blocked by a dominant-negative form of murine Nfkbia, demonstrating their involvement in notochord development of zebrafish (67). Surprisingly, in the subsequent decades, only a few substantial analyses of teleost NF-κB/Rel proteins and their functions in fish have been conducted (68–71). This is particularly striking because NF-κB/Rel-based assays, and particularly reporter-gene analyses, are widely used to assess immune stimulation, signalling activity and pathway output (72–74). Only a few studies have directly linked reporter activation to specific Rel family members, with evidence mainly from *rela* overexpression in HINAE cells from Japanese flounder (*Paralichthys olivaceus*) (71) and recent analyses of Rela and Relb in spotted sea bass (*Lateolabrax maculatus*) (75). The limited interest in characterising teleost NF-κB/Rel factors may partly due to their relatively modest transcriptional responses to infection or immune stimulation observed in several fish models so far (76, 77).

NF-κB/Rel activity is tightly regulated by multiple signalling mechanisms (**Figure 2A**) and research in fish has focused more extensively on these regulatory components than on the NF-κB/Rel factors themselves. A prominent example is the inhibitor of NF-κB (IκB) family, which retains the NF-κB/Rel dimers in the cytosol in an inactive state (67, 69, 80–83). IκB proteins are degraded following phosphorylation (84, 85) leading to activation of NF-κB/Rel signalling. In contrast, NF-κB-inhibitor-interacting Ras-like proteins (NKIRAS) stabilise the NF-κB–IκB interaction and thereby negatively regulate NF-κB/Rel activity in both mammals (86) and in fish (87) (**Figure 2A**). Additional factors that modulate NF-κB/Rel activity in fish include PIAS (protein inhibitors of activated STAT) (74) and three paralogues of the paracaspase MALT1 (mucosa-associated lymphoid tissue lymphoma translocation protein 1) (88, 89). The conservation of NFKBIA, NKIRAS, PIAS and MALT protein functions across vertebrates underscores the importance of maintaining the delicate balance between NF-κB-mediated pathogen defence and the risk of pathophysiological consequences (**Figure 2A**).

**Figure 2:**
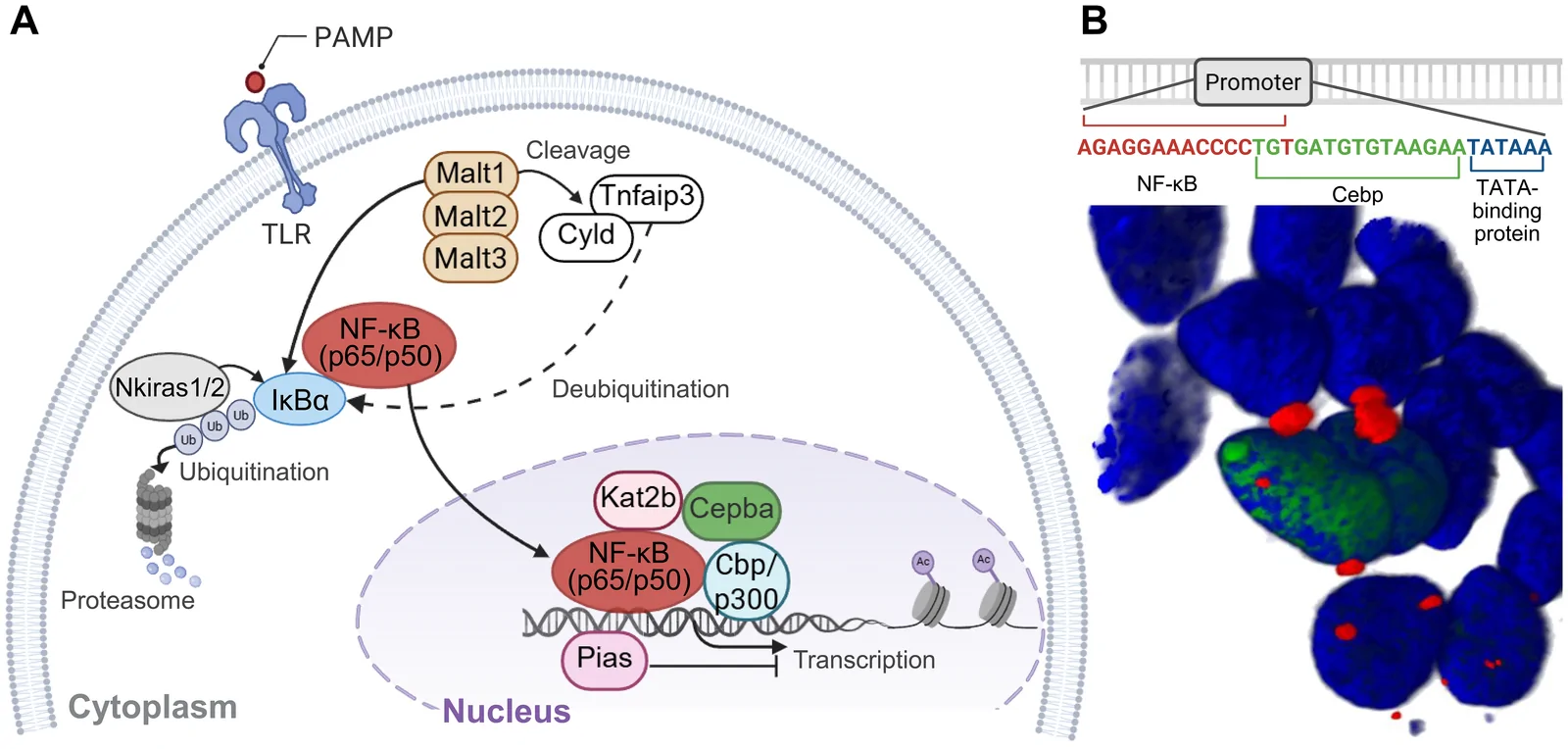
**(A)** Spatial regulation of NF-κB signalling by cytoplasmic and nuclear cofactors. Nkiras proteins interact with IκB-family members to impede their degradation, thereby stabilising the inhibitory IκB–NF-κB complex and retaining NF-κB in the cytoplasm. Upon stimulus-induced release and nuclear translocation of NF-κB, PIAS proteins negatively regulate NF-κB–dependent gene expression by repressing its transcriptional activity in the nucleus. In contrast, the transcriptional co-activators Cbp/p300 and Kat2b promote NF-κB–mediated transcription through histone and NF-κB acetylation. Malt proteins act as positive regulators of NF-κB signalling by proteolytically cleaving NF-κB inhibitors such as Tnfaip3 and Cyld, which normally promote deubiquitylation of IκBα and thereby limit NF-κB activation. **(B)** Incipient cooperation of Cebpa (green) and the NF-κB subunit Rela/p65 (red) in rainbow trout. HEK-293 cells overexpressing *cebpa* fused to green fluorescent (78) were treated with a rabbit monoclonal anti-p65 (D14E12, Cell Signaling Technology); nuclei were counterstained with Hoechst 33342. Confocal imaging shows that Cebpa is pre-bound in the nucleus, whereas NF-κB requires stimulus-dependent nuclear translocation. As promoters of teleost early response genes, such as the IER *cxcl8* (78) or the DPRG *il6* (79) frequently contain composite or partially overlapping NF-κB sites adjacent to C/ebp motifs (*cf*. promoter section above the confocal image), Rela and Cebpa rarely act independently but instead engage in functional cooperation. Created with BioRender.com.

The function of NF-κB/Rel factors is not limited to the immediate early phase of infection; rather, they act as gatekeepers that render target-gene promoters transcriptionally competent (90) and allow for an initial, moderate level of transcription. At later stages, class-II transcription factors, such as members of the C/EBP family, are recruited (**Figure 2B**) (91, 92) to stabilise the transcriptional complex and promote robust and sustained gene expression. This sequential recruitment establishes a temporally coordinated inflammatory response that is essential for tissue-specific and prolonged acute-phase reactions. NF-κB/Rel factors are therefore pivotal in shaping the direction of the immune response, and further investigation of their orthologues in fish represents a valuable avenue for advancing our understanding of early immune regulation.

#### 2.1.2 Nfat—coordinator of calcium-dependent immune activation

Structural analyses revealed that NF-κB shares similar DNA-binding domains with NFAT (93). Five NFAT proteins have been identified in mammals and are encoded by an equivalent number of *NFATC* genes (47). Contrary to what their name suggests, ‘nuclear factors of activated T cells’ are expressed in many different cell types and tissues. In resting cells, they are highly phosphorylated, whereas the calcium-dependent activation of calcineurin triggers dephosphorylation, exposing a nuclear localisation signal and enabling nuclear translocation (94). NFAT2 (encoded by *NFATC1*), NFAT1 (*NFATC2*), NFAT4 (*NFATC3*) and NFAT3 (*NFATC4*) act as monomers that directly cooperate with other transcription factors, forming multi-protein transcriptional complexes that integrate calcium signals with other signalling pathways (95). In contrast, NFAT5 is structurally more closely related to NF-κB and functions as a dimer (96).

It has been known for nearly 25 years that orthologues of all five NFAT factors are present in teleosts such as pufferfish (97). Interestingly, four paralogues of only *nfat5* were identified in Atlantic salmon (*Salmo salar*) (98) and four different Nfat proteins were apparently detected in the channel catfish (*Ictalurus punctatus*), although these may be differently phosphorylated variants rather than paralogues (99).

The expression of *nfatc3* is induced following stimulation with yeast and its PAMP zymosan of head kidney cells and *Vibrio parahaemolyticus* stimulation of head kidney lymphocytes in red snapper (*Lutjanus peru*) (100). Functional evidence from channel catfish further indicates that Nfat activity is regulated at the level of calcineurin-dependent dephosphorylation and nuclear translocation: ciclosporin inhibits concanavalin A-induced leucocyte proliferation when applied before, but not after, activation has been initiated, whilst phosphatase assays and EMSA demonstrated inhibition of Nfat dephosphorylation and Nfat binding to its cognate response element, respectively (99). Together, these findings suggest that Nfat signalling contributes to the early transcriptional response to immune stimulation in teleost leucocytes and the importance of Nfat for T-cell proliferation in bony fish. Nevertheless, the induced *nfatc3* expression does not indicate that Nfatc3 itself is a primary regulator of immune-gene transcription or reflects downstream activation of the pathway. Time-resolved perturbation of Nfat combined with genome-wide analyses of binding characteristics and chromatin accessibility would help distinguish direct transcriptional targets from secondary responses.

#### 2.1.3 Irf—master regulators of interferon responses

The IRF family comprises more than 10 members with diverse functional roles in the type-I IFN pathway (101). This evolutionarily ancient family (102) diversified before the divergence of tetrapods and teleosts, with several IRF members sharing orthologues even in invertebrates (103). IRF1 and IRF2 were the first members of the family to be identified in mammals (104, 105). Both bind the same regulatory motif in IFN promoters, with IRF1 acting as a transcriptional activator and IRF2 as a repressor (106).

In salmonids, 23 Irf family members were identified reflecting extensive gene retention after additional genome-duplication events (107). The genes encoding Irf1 and Irf2 were first isolated in a salmonid species by homology cloning in 2003 (108) and were subsequently recognised as *irf1–2* and *irf2-2*, respectively, within the expanded salmonid Irf1/2 repertoire (107). With the increasing availability of ESTs and genomic data, Irf3 to Irf10 were progressively identified and functionally characterised in various teleosts, with the majority of studies concentrating on Irf3 and Irf7 (109).

IRF3 and IRF7 are two critical members of the IRF family, as they are involved in the initial production of IFNs in response to viral infection. In vertebrates, viral sensing through endosomal TLRs (**Figure 1**) and cytosolic receptors (110, 111) can activate IRF3 and/or IRF7 through phosphorylation-dependent mechanisms, followed by homo- or heterodimerisation and nuclear translocation. The relative contribution of individual PRR–adaptor pathways, however, varies amongst teleost species. Structural studies of IRF3 revealed that its N- and C-terminal autoinhibitory regions fold together to form a hydrophobic surface that masks the IRF association domain and prevents the interaction with other proteins. Upon activation, phosphorylation induces a conformational rearrangement of the autoinhibitory regions, allowing IRF3 to dimerise and initiate downstream signalling (112).

The role of IRF7 is more complex. In mammals, IRF7 has a short half-life and its expression is largely restricted to specific immune cell types, where it is either constitutively expressed or strongly induced by type-I IFNs (113, 114). Unlike IRF3, IRF7 preferentially activates a distinct subset of type-I IFN gene promoters (115). Furthermore, the potential formation of IRF3–IRF7 heterodimers and the existence of four alternatively spliced IRF7 isoforms point to a highly complex regulatory network that has yet to be fully elucidated (113, 116). Type-I IFNs activate the Janus kinase (JAK)–STAT pathway, inducing hundreds of IFN-stimulated genes (ISGs), including *IRF1*, *STAT1* and *IRF7*. These transcriptional regulators reinforce IFN signalling through positive feedback whilst driving a broad antiviral gene programme. Accordingly, the canonical antiviral response comprises two phases: an initial PRR–IRF3–IFN signalling phase, followed by IFN–JAK–STAT-mediated induction of more than 200 ISGs. Amongst these, IRF1 is a key regulator of the secondary IFN response, sustaining antiviral gene expression.

In salmonid fish, analyses of newly assembled genomes have revealed that, unlike *irf3*, two paralogues of *irf7* have been retained (117, 118), corresponding to the same paralogue *irf7.1* (107). Recently, it was demonstrated that PRR signalling is mediated predominantly through Irf3 (119), whereas the specific functions of the two *irf7* paralogues (*irf7.1* and *irf7.2*) remain elusive. More broadly, the distinct roles of Irf3 and Irf7 in teleosts remain underexplored. An earlier EMSA study failed to detect specific binding of either salmonid Irf3 or Irf7.1 to the *mx1* promoter, suggesting that the repertoire of genes regulated by Irf3/Irf7 may differ from the ISGs controlled by the ISGF3 complex (117). Integrating gene-knockout approaches with epigenomic analyses (30) will further elucidate the promoter-binding landscapes and transcriptional targets of Irf3 and Irf7 paralogues. Consistent with this, loss-of-function studies in salmonid cell systems have shown that disruption of key antiviral signalling components has only limited effects on viral resistance (120–122), underscoring the redundancy and robustness of the Ifn system. These findings highlight the need for integrative approaches to unravel the complex regulatory architecture of antiviral immunity.

### 2.2 Level II: transcription factors driving secondary responses

Class-II transcription factors are newly synthesised in response to appropriate signalling cues and subsequently accumulate in the cell nucleus. They are often encoded by IEGs whose expression is induced by class-I transcription factors (39) (**Figure 1**). To close the circle, class-II transcription factors often interact with class-I transcription factors at the promoters of secondary response genes (**Figure 2B**), often contributing to feed-forward loops and autoregulatory transcriptional circuits (91, 123).

Class-II transcription factors are much more extensive than those of class I and include a remarkable number of immune factors that adapt immune programmes specifically to a given stimulus and, in some cases, even counteract responses that were previously triggered by class-I transcription factors (124–126). They are usually effective for longer periods of time, but are also subject to feedback loops that control their expression. Genes regulated by either class-I- or class-II transcription factors can be identified experimentally by using protein-synthesis inhibitors, such as the antibiotic cycloheximide (127), which block the production of class-II factors. In addition, transcriptome analyses can detect the stimulus-dependent regulation of the transcript concentration of class-II transcription factors and the genes induced by them (128).

Amongst the best-characterised class-II transcription factors in fish are the pleiotropic ‘CCAAT/enhancer-binding proteins’. In contrast, teleost AP-1 factors and the larger ‘signal transducers and activators of transcription’ (Stat) family remain less well understood. The following subsections provide a more detailed overview of these and other class-II transcription factors and their roles in fish immunity.

#### 2.2.1 The multifaceted AP-1 factor—integrator of Mapk signalling

Inflammatory and stress signals regulate the defined sequence of various ‘mitogen-activated protein kinases’ (MAPK) via a cascade of phosphorylation events. These kinases activate the ‘extracellular signal-regulated kinase’ (ERK), the ‘c-Jun N-terminal kinase’ (JNK) and the p38 kinase, which ultimately activate the activator proteins 1 (AP-1) (129). The MAPK-mediated activation of transcriptionally competent AP-1 has likewise been demonstrated in fish (130, 131). AP-1, similar to NF-κB, does not denote a single transcription factor (132) but rather a group of dimeric transcription factor complexes that are also present in teleosts: Jun (Jun, Junb and Jund), Fos (Fos, Fosb and Fosl1a/b), musculoaponeurotic fibrosarcoma factor (Maf with Mafa, Mafb, Mafga/b, Maff, Mafk and Nrl) and the activating transcription factors (Atf with Atf2, Atf3, Batf and Jdp2a/b (133–135). Some of these AP-1 components are pre-existing (and become activated as primary transcriptional regulators), whereas others, including Junb, Jund, Fosb, Mafb, Maff, Atf3, Batf and Jdp2a/b, are synthesised as typical IEG products and often confer enhanced or repressive transcriptional activity, depending on the dimer composition and cellular context.

Upon appropriate stimuli, Atf, Maf, Jun and/or Fos proteins form homo- or heterodimers via their basic leucine zipper (bZIP) domains. The resulting AP-1 complexes bind to 12-O-tetradecanoylphorbol-13-acetate (TPA)-responsive elements (TREs) (136) as well as related cAMP response element (CRE)- and MAF-recognition element (MARE)-like motifs (137, 138), depending on their dimer composition. As a prototypic example, members of the Fos family heterodimerise with members of the Jun family to form transcriptionally active AP-1 complexes that bind their cognate TREs present in many promoters and enhancers of genes encoding cytokines and other inflammatory mediators, stress-responsive and apoptotic genes or genes related to cell cycle and proliferation (139, 140).

Evidence from teleosts supports AP-1 as a broadly conserved transcriptional module linking immune signalling to changes in gene expression, but its regulation appears to be highly context-dependent. Reporter-gene assays demonstrated that *jun* and *fos* from red lip mullet (*Liza haematocheila*) act cooperatively to enhance AP-1 promoter activity (141), whilst functional studies of Tlr signalling revealed opposing effects: Tlr8 from common carp (*Cyprinus carpio*) activates AP-1 (142), whereas Tlr22 from orange-spotted grouper (*Epinephelus coioides*) suppresses NF-κB activity whilst selectively activating MAPK signalling, which, in turn, reduces AP-1 activity (143). The additional induction of genes coding for AP-1 components, including *jun*, *fos*, *fosb*, *fosl2* and *jund*, following viral infection or bacterial stimulation (130, 141, 144) indicates that AP-1 activation cannot simply be regarded as a uniform downstream response to PAMP recognition. Rather, its transcriptional output may depend on the upstream cell type and its receptor repertoire, as well as on the type and duration of stimulus exposure.

Consistent with such context dependence, AP-1 components also participate in light-responsive, circadian and regenerative programmes in zebrafish (145, 146). Although transcriptomic studies frequently identify *jun*, *fos*, *maf* and *atf* genes as components of immune-associated signatures (147–149), differential expression alone does not establish, which AP-1 complexes are functionally active or which target genes they directly regulate. A key unresolved question is therefore how different combinations of AP-1 components integrate distinct upstream signals to generate stimulus- and cell-type-specific transcriptional responses. This could be addressed by combining targeted perturbation of individual AP-1 components with time-resolved chromatin-binding and transcriptomic analyses to distinguish direct AP-1 targets from secondary changes in gene expression.

Unlike some AP-1 subunits such as FOS or JUN, which are often constitutively expressed and await their nuclear translocation, *ATF3* may exhibit high fold inductions, facilitating its detection by various techniques (150), also in fish (128). This makes *ATF3* one of the ‘first-pass’ genes for screening immune or stress responses, especially in non-model species (151). High *Atf3* expression has been reported in the head kidney of Nile tilapia (*Oreochromis niloticus*) (135), whilst its expression increases approximately twofold in zebrafish within a few hours of *Citrobacter freundii* infection (152) and by more than 60-fold in the spleen of Atlantic cod (*Gadus morhua*) at a similar time after poly(I:C) stimulation (153). ATF3 is well conserved across vertebrates (154) and responds to a variety of stimuli, which are mainly mediated by various TLR signalling cascades (155). Functioning both as a homodimer and as a heterodimer with ATF2, JUN or JUNB (156), ATF3 can repress the expression of numerous genes that encode inflammatory cytokines, such as interleukin-6 (IL6) (155). Alternatively, ATF3 can also inhibit the expression of *CEBPD*, whose gene product C/EBPδ is essential for IL6 synthesis (157).

Similar to NF-κB/Rel factors, it is striking that the scope of existing studies on the various teleost AP-1 subunits, or on their individual functions during immune responses, does not reflect their apparent importance in health and disease. Consequently, the frequently invoked—yet often insufficiently nuanced—call for more comprehensive and in-depth research is fully justified in this context.

#### 2.2.2 Creb—a potent integrator of second-messenger signalling

The CREB family comprises numerous transcription factors that can act either as activators or repressors, depending on their isoform and promoter context. Members of the CREB family (CREB1, CREB3, CREB5, as well as CREB3L1, CREB3L2, CREB3L3 and CREB3L4) and CREM (cAMP response element modulator) are, similar to ATF proteins, characterised by a transactivation domain and a dimerisation domain and are activated by phosphorylation through cAMP-dependent serine/threonine kinases (158). Both CREB and ATF proteins bind via their leucine-zipper motif to a common palindromic CRE motif (159). Consequently, heterodimers composed of CREB and ATF can bind to this element, which is found, for example, in the promoters of various cytokine-encoding genes (160).

Whereas mammalian CREB1 is activated immediately following stimulation of PRRs, B-cell receptors and T-cell receptors (161–163), CREB3 and CREB5 are involved in longer-term adaptive and differentiation programmes (164). These CREB proteins are initially anchored in the ER membrane and are activated in response to specific signals through regulated proteolytic cleavage (164). CREB1 promotes maximal transcription of its target genes within 30 minutes of activation, whereas methylation of the CRE motif impairs CREB binding (165). In mammals, CREB factors regulate cell proliferation and differentiation and contribute to immune responses (158). Here, they mainly promote anti-inflammatory functions by inhibiting NF-κB signalling through competition for the co-activators CREB-binding protein (CBP)/p300 (166) or by inducing the expression of the anti-inflammatory cytokine *IL10* (160). CREB also supports regulatory T-cell differentiation as part of the c-Rel enhanceosome (167).

Similar to several *atf* genes, *creb1*, *creb5* and *crem* are present as multiple paralogues in some fish species, such as the common carp (135). This gene multiplication may reflect comprehensive sub-functionalisations, as Creb factors in fish are involved in a wide range of biological processes. Nonetheless, relatively little is known about the Creb-dependent regulation of immune responses in fish: a study in sea bream (*Sparus aurata*) demonstrated that prostaglandin PGE2 induces phosphorylation of Creb and Atf1 in macrophages, promoting their differentiation towards the anti-inflammatory M2 phenotype (168).

CREM represents a particularly interesting factor in mammals. On the one hand, it can act as an early-response activator; on the other hand, specific alternatively spliced isoforms (e.g., ICER) function as transcriptional repressors during later stages of an immune response (169). Although CREM orthologues remain poorly characterised in fish, available evidence suggests their contribution to distinct transcriptional signatures. For instance, they have been implicated in spawning male bluegill sunfish (*Lepomis macrochirus*) (170) and in fathead minnows (*Pimephales promelas*) exposed to polycyclic aromatic hydrocarbons (171). These observations highlight CREM orthologues as promising candidate genes for future studies investigating immune competence during the spawning season and in response to environmental stress.

#### 2.2.3 The versatile and pleiotropic C/ebp family

CCAAT/enhancer-binding proteins (C/EBPs) are leucine zipper transcription factors comprising six members in mammals: C/EBPα, C/EBPβ, C/EBPγ, C/EBPδ, C/EBPϵ and C/EBPζ encoded by the genes *CEBPA*, *CEBPB*, *CEBPC*, *CEBPD*, *CEBPE* and *CEBPZ*, respectively (172, 173). These genes are expressed in a wide variety of cell types, reflecting their involvement in diverse biological processes, including immunological, haematopoietic and metabolic functions. C/EBPs contain domains responsible for transcriptional activation or repression, dimerisation and DNA binding, and individual family members can partially compensate for one another (174).

Teleost orthologues of the *CEBP* genes *A*, *B*, *C*, *D* and *E* were first characterised in zebrafish (175) and Japanese flounder (176). A teleost orthologue of *cebpz* has not yet been functionally characterised; nevertheless, annotated *cebpz* sequences are present in the NCBI databases and transcripts have been detected in the transcriptional signatures of temperature-challenged rainbow trout (*Oncorhynchus mykiss*) (177). Several paralogues of Cebp-encoding genes have been identified in the rainbow trout, including duplicated *cebpb* and *cebpd* genes (178). In addition, numerous transcript variants exist and, as in mammals, shorter Cebpb isoforms in rainbow trout compete with longer variants for binding to the same response elements (179). Besides the six C/EBP factors apparently conserved across vertebrates, a presumably fish-specific Cebp1 variant has been identified in zebrafish. *Cebp1* appears to be expressed mainly in cells of myeloid origin and is involved in the regulation of a lysozyme-encoding gene (180, 181).

Teleost Cebp factors appear to have conserved functions in haematopoiesis, but their precise roles in immune regulation remain less clearly defined. Cebpa and Cebpb orthologues are expressed in haematopoietic cells (175, 178, 182), although differences in subcellular localisation have been reported between teleost and mammalian C/EBP proteins (78). Functional studies in zebrafish highlight their importance in haematopoiesis: the activation of Cebpa promotes myelopoiesis from myelo-erythroid progenitor cells during primitive haematopoiesis (183), whilst a truncated Cebpa variant triggers the expansion of primitive erythroid cells in the head kidney of zebrafish embryos without strongly impairing granulocyte differentiation (182). Genetic inactivation of *cebpa* likely disrupts the differentiation of myeloid precursor cells into macrophages and granulocytes in zebrafish embryos (184). These findings support Cebpa as an important regulator of myeloid-cell development, but they do not necessarily indicate that the same mechanisms govern Cebp-dependent transcription in mature immune cells.

The relevance of Cebp factors for immune responses in fish is also reflected by the presence of Cebp-response elements. These are–frequently located in close proximity to NF-κB binding sites (**Figure 2B**)–present in numerous key immune genes of bony fish, including those encoding C-type lectin receptors (185), inflammatory cytokines such as Cxcl8 and Il6 (78, 79), acute-phase proteins or MHC molecules (12). Consistent with this regulatory potential, *cebpa* or *cebpb* expression increases following LPS stimulation or bacterial and viral infection in several teleost species, including rainbow trout, Japanese flounder, mandarin fish (*Siniperca chuatsi*) and zebrafish (186–190).

Nevertheless, these expression patterns are not sufficient to conclude that teleost Cebp factors directly drive the observed immune-gene responses; alternatively, Cebps may themselves be induced as part of broader inflammatory programmes. Moreover, the functional significance of potentially overlapping Cebpa- and Cebpb-dependent regulation remains unresolved. An important next step would therefore be to determine whether individual Cebp factors directly occupy immune-gene regulatory regions and whether their perturbation alters NF-κB/Rel-dependent transcription. Combining Cebp-specific loss- or gain-of-function approaches with chromatin-binding and transcriptomic analyses could distinguish direct Cebp targets from secondary responses and clarify whether Cebp factors provide distinct regulatory functions during teleost immune activation.

Epigenetic and post-translational mechanisms contribute to Cebp regulation in teleosts. In zebrafish, expression of a truncated variant of the *dnmt1* gene, encoding DNA methyltransferase 1, reduces DNA methylation at specific genomic regions, likely including the *cebpa* promoter and thus leading to abnormal haematopoiesis (191). Cebp activity is also modulated by acetylation and phosphorylation in rainbow trout (Lo and Chen, 2010), SUMOylation in zebrafish (183) and microRNAs such as microRNA-27b, which enhances *cebpa* expression (192).

Altogether, teleost Cebp factors regulate a wide range of physiological processes, including inflammatory responses and immune cell differentiation. As such, they are considered versatile regulators that act in concert with NF-κB/Rel or Stat factors and other transcriptional regulators to maintain homeostasis and mediate responses to disruptive external influences in fish.

#### 2.2.4 Stat—drivers of cytokine-activated gene expression

TLR and other innate-receptor signalling pathways prominently activate NF-κB/Rel factors, whereas cytokine receptors predominantly activate STAT proteins. These pathways nevertheless converge extensively and can cooperate at shared target genes. These ‘signal transducers and activators of transcription’ form an evolutionarily ancient family comprising seven members (102, 193). Teleost fish possess all STAT members described in humans (194, 195). Amongst these, Stat1 and Stat2 are central to IFN signalling, the precise mechanisms of which have been well characterised in humans (46) and recently validated in a salmonid *in vitro* model, where Stat2 was shown to be involved in type-I but not type II-IFN signalling (122).

The combination of different STAT proteins into homo- and heterodimers, together with their association with Jak/Tyk kinases, defines the downstream response (196, 197). Apart from STAT2, there is limited evidence that fish follow the canonical pathway described in human and mouse models. Unlike *stat2*, which exists as a single copy in all fish species, *stat1* has been retained as two genes in teleosts, with further expansion to four or even five *stat1* paralogues in salmonids (198). In mammals, *STAT1* and *STAT2* are each encoded by single genes; however, two alternatively spliced *STAT1* isoforms have been described (199) and multiple isoforms are annotated in current genome assemblies.

This variability may represent a host strategy to counteract viral immune evasion mechanisms (200) highlighting the functional versatility of the Stat family in fish, which likely enables a finely tuned, context-dependent regulation of antiviral and immunological responses. Whereas Stat1, Stat2 and Stat3 are key regulators of the early immune response, promoting the induction of ISGs and the establishment of antiviral defence programmes, the other Stat family members (Stat4, Stat5 and Stat6) primarily contribute to adaptive immunity by regulating lymphocyte differentiation and lineage-specific immune functions (198, 201, 202). For further details on the evolutionary origin, diversification and immune-related functions of Stat factors in fish, we refer to comprehensive reviews by Boudinot et al. (198) and Wang & Levy (203).

### 2.3 Level III: cell type-specific transcription factors for specialised defence mechanisms

A third class of immune-relevant transcription factors primarily establishes and maintains cellular identity rather than mediating acute immune responses. Unlike primary and secondary transcription factors that control broadly inducible mechanisms, transcription factors of class III operate in a cell type-restricted manner (**Figure 1**), specifying lineage-dependent gene-expression programmes that restrict immune functions to defined cellular subsets (204).

The organisation of the teleost immune system provides an important anatomical and cellular context for understanding these lineage-specific transcriptional programmes. Unlike mammals, teleosts lack bone marrow and lymph nodes; instead, the head kidney serves as a major site of haematopoiesis and immune-cell development (205). Together with the spleen, the head kidney provides the adequate environment for proliferating and differentiating B- and T-cell populations (206–208). Consequently, transcriptional programmes that in mammals are often associated with bone-marrow-derived progenitors or organised lymphoid tissues are distributed across multiple anatomical compartments in teleosts. This is particularly relevant when interpreting tissue-level expression of immune transcription factors, because the head kidney functions both as a site of immune-cell development and as an important effector organ (205). In addition, mucosal tissues, including the gills, intestine and skin, represent major sites of pathogen exposure and immune surveillance (209). Lineage-associated transcription factors are therefore detected across both systemic and mucosal compartments (210), highlighting the importance of considering tissue-specific cellular composition, developmental stage and local environmental exposure.

At the cellular level, commitment of haematopoietic progenitors to the lymphoid lineage depends on a balance between transcriptional programmes promoting lymphoid and myeloid development. The zinc-finger transcription factor IKAROS (*IKZF1*) initiates this process and is required for lymphoid priming and maintenance of early lymphoid progenitor states across vertebrates (211–216). In teleosts, Ikaros contributes not only to lineage priming but also to downstream branching events within the B-cell lineage, thereby coordinating the assembly of antigen-receptor genes during early differentiation (217). Opposing this lymphoid programme, the factor SPI1 (encoded by the ‘spleen focus-forming virus integration 1’ gene) acts as a conserved regulator of myeloid fate determination and innate immune specification, functioning as a key dose-dependent switch between myeloid and lymphoid trajectories (218, 219). Functional studies in cartilaginous and teleost fish have primarily defined PU.1 activity in macrophage development and inflammatory responses (180, 187, 220–222), whilst teleost-specific gene duplications further refine inflammatory and phagocytic programmes (218). Successful lymphoid commitment consequently requires attenuation of PU.1 activity (223).

Following lineage commitment, class-III transcription factors contribute to the specification and functional differentiation of individual immune-cell populations. In B cells, for example, these programmes support the development of antibody-producing populations responsible for humoral defence mediated by the three major teleost immunoglobulin classes, IgM, IgD and IgT (224–227). T-cell differentiation likewise involves conserved lineage-associated transcription factors, although the extent to which the canonical mammalian T-cell subsets are conserved as discrete and functionally equivalent populations in teleosts remains an active area of investigation (see Section 2.3.2). B- and T-cell programmes are further integrated through reciprocal cellular interactions, whilst additional lymphocyte populations, including natural killer cells, contribute to immune surveillance through receptor-mediated recognition of altered or missing self-markers (228).

Importantly, class-III factors do not function independently of the broader immune transcriptional network. Rather, lineage-specific programmes are integrated with inducible class-I and class-II transcription factors, including Stats and Irfs, which enable differentiated immune cells to respond dynamically to cytokines, pathogens and other environmental signals (229–232). Thus, transcriptional regulation in teleost immune cells emerges from the interaction between relatively stable lineage-defining programmes and inducible transcriptional responses. A considerable number of additional transcription factors contribute to these networks, of which selected examples are discussed in the subsections below.

#### 2.3.1 PU.1—master regulator of hematopoietic lineage specification

Members of the small PU.1 (purine box 1) family belong to the ETS (E26 transformation-specific) transcription factors, which are almost exclusively present in haematopoietic cells (233). The PU.1 factor (SPI1) controls the development of macrophages, granulocytes, T and B cells in mammals, whilst its paralogues SPIB and SPIC are more specifically involved in B-cell development (234). PU.1 acts as a pioneer factor (235) recruiting cell type-specific activators and protein complexes that methylate H3 histones at relevant enhancer regions, thereby rendering the chromatin accessible to other transcription factors (236).

The first *spi1*-like sequence identified in a fish species was reported in the sea lamprey (*Petromyzon marinus*). Although it shares relatively low sequence similarity to the mammalian orthologous gene, *spi1* is nevertheless expressed in lymphocyte-like cells of the digestive tract (237). In a representative of the cartilaginous fish, the clear-nosed ray (*Raja eglanteria*), *spid* was discovered in addition to *spi1* and *spib*, which are both present in mammals. *Spi1* and *spid* are mainly detectable in gonads and Leydig organs, which contain B cells and myeloid cells but hardly any T cells. By contrast, *spic* expression is predominant in the T-cell–containing spleen (220).

Two copies of the mammalian *SPI1* orthologue have been characterised in various model-fish species (218, 238), but most of the knowledge about teleost Spi1 factors has been gathered in studies on zebrafish. The expression of *spi1* is detectable within a few hours after fertilisation (239) and contributes to the speciation of haematopoietic lineages (240). Various constructs in which the promoter of a zebrafish *spi1* gene was coupled to green fluorescent protein (GFP) have been used to generate transgenic lines of myeloid cells visible in almost transparent zebrafish embryos (31, 241). These models have revealed key mechanisms controlling cell differentiation during embryonic myelopoiesis and erythropoiesis. Spi1 and Gata1 act as opposing lineage determinants, directing progenitors towards myeloid and erythroid fates, respectively, through a mutually antagonistic bistable regulatory circuit (242). Additional factors, including Cebp1 and Runx1, regulate granulocyte maturation, whereas Spi1 is required for macrophage development (180, 243). Thus, lineage commitment depends on transcription factor dosage and feedback regulation rather than simple binary switches. Beyond haematopoietic differentiation, Spi1 also cooperates with Cebpa to regulate immune responses, as shown by their joint activation of *tnf* expression in Japanese flounder HINAE cells after LPS stimulation (187).

In embryonic zebrafish, *spi1* is expressed in the brain-resident macrophages, known as microglia, and its deficiency leads to the progressive loss of these cells (244, 245), supporting a role in microglial maintenance rather than merely serving as a lineage marker (222). Consistent with this broader role in macrophage biology, Ifng induces *spi1a* and *spi1b* expression in macrophage cells of yellow croaker, where both factors can bind the promoter of *cathepsin S* and promote its expression (238). However, evidence from ayu (*Plecoglossus altivelis*) indicates that the two paralogues are not functionally equivalent: *spi1a* and *spi1b* are both induced early following *Vibrio anguillarum* infection and translocate to the nucleus, yet knockdown of *spi1a*, but not *spi1b*, alters the expression of selected pro- and anti-inflammatory cytokines. Conversely, depletion of *spi1b* increases bacterial survival, suggesting that this paralogue may contribute more specifically to antibacterial defence (218). Thus, similar expression patterns do not necessarily imply redundant functions, and the distinct effects of the two paralogues point to differential regulatory outputs downstream of Spi1.

The above finding supports Spi1 as an important regulator of myeloid-cell identity and function in teleosts, whilst also revealing functional divergence between its paralogues. Differences in DNA-binding specificity, interaction partners, subcellular dynamics or target-gene selection could allow Spi1 paralogues to regulate partially distinct transcriptional programmes, but this has not yet been systematically tested in teleosts. Additional regulatory complexity may arise from non-coding mechanisms: antisense long non-coding RNAs (lncRNA) targeting *spi1* can modulate immune-relevant gene expression in zebrafish, with effects opposite to those observed following *spi1* overexpression (246, 247). However, whether these lncRNAs directly regulate Spi1-dependent transcription or act through broader effects on cellular differentiation remains unresolved. Comparative perturbation of *spi1* paralogues combined with genome-wide binding and transcriptomic analyses would therefore provide a means to determine whether their apparent functional divergence reflects distinct genomic targets or differences in the cellular context in which Spi1 activity is deployed.

In mammals, SPI1 also acts as a developmental gatekeeper in early T-cell progenitors and as a fine-tuner of helper T-cell (T_H_) differentiation, primarily by repressing T_H_2 lineage programmes (248). In teleosts, however, lymphoid lineage regulation has been studied primarily through other key transcriptional regulators, including T-bet and EOMES.

#### 2.3.2 Orchestrators of T-cell identity and differentiation

T-cell lineage specification in fish involves several transcriptional regulators that are conserved across vertebrates (249), but the extent to which the canonical mammalian T-cell subsets are conserved as discrete and functionally equivalent cell populations in teleosts remains unresolved (250–253). Accordingly, terms such as T_H_1-, T_H_2-, T_H_17- and T_reg_-like cells are generally used to describe transcriptional or functional states inferred from the expression of lineage-associated markers and transcription factors rather than to denote definitively established homologous T-cell lineages. Key lineage-associated factors include T-bet/Stat1 and Stat4 in T_H_1-like responses (254), Gata3/Stat3, Stat5 and Stat6 in T_H_2-like responses (255), Rorc/Stat3 and Stat4 in T_H_17-like responses (256), and Foxp3/Stat3 and Stat5 in regulatory T-cell functions (251) (**Figure 3**).

**Figure 3:**
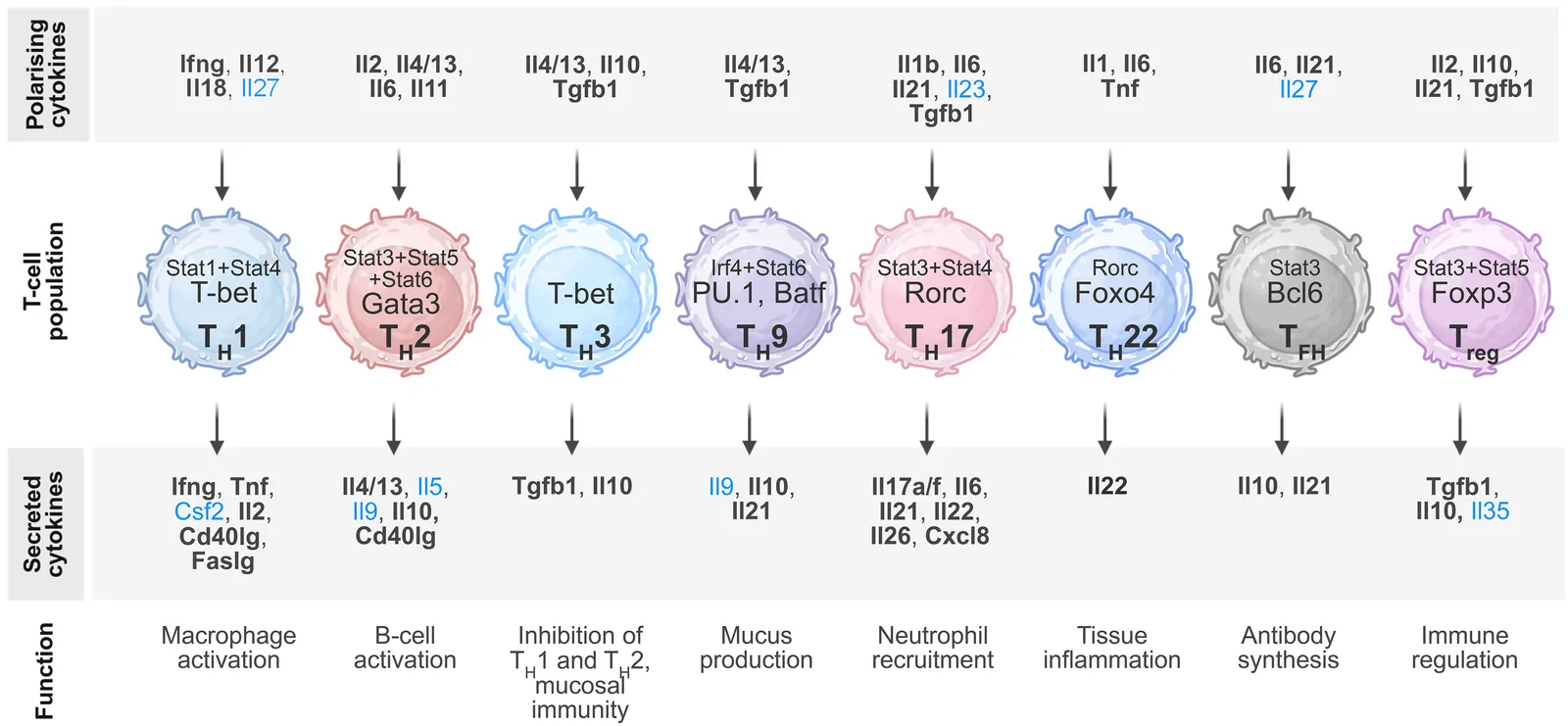
Cytokines and transcription factors controlling the polarisation of vertebrate T helper (T_H_) cell populations. Lineage-specific transcription factors translate the received cytokine signals into a differentiation programme. Note that only most characteristic representatives of the relevant transcription-factor repertoire are depicted. The resulting T-cell populations in turn secrete different sets of cytokines to trigger specific immune functions. Cytokines that have only been identified in mammalian species are marked in blue; evolutionarily conserved cytokines from fish to humans are highlighted in bold. The figure illustrates that most of the putative polarisation signals are present in fish, although T_H_-cell polarisation in fish remains to be functionally explored (257). Created with BioRender.com.

The lineage-determining transcription factors of class III control cellular identity not only through direct regulation of gene expression but also by shaping chromatin accessibility. Pioneer factors such as PU.1 bind to deacetylated enhancer regions and initiate chromatin restructuring, thereby facilitating the recruitment of additional transcription factors (236). The spatial proximity between enhancer and promoter regions is typically achieved through the formation of chromatin loops. Similar epigenetic mechanisms are increasingly recognised in teleost immune regulation, although their contribution to T-cell differentiation and the establishment of stable T-cell states remains less extensively characterised compared to mammals (29).

##### 2.3.2.1 T-bet and Eomes—linkers between T-boxes and T-cell fate

The ancient family of T-box transcription factors comprises 17 members in humans (258), which contain the eponymous T-box. Whilst members of this family are indispensable for numerous developmental processes (258), T-bet (T-box expressed in T cells) and EOMES (eomesodermin) are of particular importance in the context of T-cell differentiation (259).

T-bet, encoded by the *TBX21* gene, influences T-cell-mediated and mucosal immune responses in mammals (210) and has already been investigated in immunological contexts in cypriniformes (202, 260, 261), salmonids (261, 262) and pleuronectoids (263). Here, *tbx21* is represented by a single orthologue that is particularly highly abundant in the spleen and head kidney (260–264). These observations indicate the pronounced importance for immune cell populations in diverse fish species and, in line with that, *tbx21* is expressed by sorted Cd4-1^+^/Cd4-2^+^ and Cd8^+^ T cells as well as IgM^+^ B cells of Japanese flounder (263). Noteworthy, one SNP within the first intron of the *tbx21* gene of Asian seabass (*Lates calcarifer*) has been identified as significantly associated with resistance to betanodavirus (265).

It has been widely proposed that intracellular bacterial and viral pathogens tend to induce T-bet–associated T_H_1-like responses, whereas extracellular pathogens, particularly helminths, often promote Gata3-driven T_H_2-like responses. The results from corresponding studies in fish have not conclusively followed this paradigm and were, in part, contradictory. Phytohaemagglutinin (PHA) induces the expression of both *tbx21* and *gata3*, whilst poly(I:C), mimicking a viral infection, reduces their expression (264). The same study found that infection with *Y. ruckeri* most likely downregulates both *tbx21* and *gata3*, whilst infection with *T. bryosalmonae* induces the expression of both genes (264). A waterborne infection with *A. salmonicida* modestly increases *tbx21* transcripts in spleen of Atlantic salmon (262).

Although previous infection studies did not allow conclusions about whether extracellular (*V. anguillarum*), intracellular (Hirame rhabdovirus virus) or facultative intracellular pathogens (*Yersinia ruckeri, Edwardsiella tarda*) trigger T-bet-driven T_H_1 differentiation (263, 264), studies on sorted Cd4^+^ cells from Japanese flounder were more informative. The stimulation with poly(I:C) induces *tbx21* expression, whereas PMA (phorbol 12-myristate 13-acetate) stimulation enhances the expression of its functional antagonist *gata3* (266). *Tbx21*-expressing Cd4-1^+^/Cd4-2^+^ and Cd8^+^ T cells from head kidney of Japanese flounder proliferate after stimulation with the cytokines Ifng and Il2 (263). Similarly, Il2 induces the expression of *stat1, ifng* and *tbx21* in lymphocytes of Nile tilapia, presumably through Stat5 and mTORC1 signalling (267). These findings, however, primarily demonstrate inducible expression and cellular activation rather than indicating the downstream genes directly controlled by T-bet. This distinction is particularly relevant because *in vivo* overexpression of salmon *tbx21* increased its own transcript abundance at the injection site but failed to confer detectable protection against bacterial challenge (268). Thus, elevated *tbx21* expression alone may be insufficient to drive effective antimicrobial immunity, suggesting that its function depends on cellular context, appropriate upstream signalling or cooperation with additional transcription factors.

T-bet, together with EOMES, guides the differentiation and maturation of mammalian CD8^+^ T cells (269). Two *eomes* paralogues were likely retained in bony fish. In zebrafish, both *eomes* paralogues are prominently expressed in leucocytes from the spleen, trunk kidney and peripheral blood, although their expression profiles differ (270). These distinct expression patterns were subsequently confirmed in two additional fish model species, rainbow trout and ginbuna crucian carp (*Carassius auratus langsdorfii*) (271). Whilst the *eomes* variant *a* is involved in establishing left–right asymmetry of internal organs in zebrafish (272), considerably less is known about variant *b*. Nevertheless, it is evident that the two genes are not co-regulated and have clearly diverged in function (273).

Two *eomes* paralogues are also present in Atlantic salmon, and at least one of them contributes to the regulation of cytotoxic and Ifng-associated programmes in salmonid lymphocytes. Following *Aeromonas salmonicida* infection, *eomes* transcripts increase in the spleen, whilst overexpression of *eomes in vitro* enhances the expression of *ifng* and the cytolytic effector gene encoding granzyme A (274). Conversely, siRNA-mediated knockdown of *eomes* reduces both *eomes* and *ifng* expression but markedly increases *tbx21* expression, suggesting compensatory regulation between these two T-box transcription factors (274). This reciprocal response is particularly notable because it indicates that Eomes activity may be buffered by T-bet rather than acting as an isolated regulator of the Ifng programme.

However, the extent to which this compensation preserves T-cell function remains unknown, and the molecular basis of the proposed Eomes–T-bet relationship has not been explored yet. Motif analysis of an *eomes* promoter fragment of more than 1700 bp from Atlantic salmon identified potential binding sites for several immune-relevant transcription factors, including Cebpb, Stat1, Ets1, Gata3 and Foxo, providing candidate mechanisms for upstream regulation but not evidence of direct binding or functional control (274). Targeted perturbation of both *eomes* paralogues and *tbx21*, ideally combined with chromatin-binding and transcriptomic analyses, would therefore be required to determine whether these factors act redundantly, compensate for one another or establish distinct effector programmes in teleost lymphocytes.

##### 2.3.2.2 Gata3—driver of T_H_2-like responses

The antagonist of T-bet during the polarisation of naïve T cells into T_H_1 cells is GATA3, which suppresses T_H_1 responses whilst promoting the differentiation of T_H_2 cells, a mechanism that appears conserved in fish (257, 266).

GATA3 belongs to the GATA family of zinc finger transcription factors, which comprises six members (GATA1–6) in mammals (275). GATA3 is a highly cell-type–specific transcription factor and a key regulator of T_H_2 lymphocyte differentiation in mammals, where it promotes the expression of *IL4* and *IL13* as well as *IL5* (201). The teleostean Il4/13 orthologue is often present in multiple isoforms, one of which contains both TATA box and Gata3-binding motifs in its proximal promoter region (276, 277).

Like *tbx21*, the teleostean *gata3* orthologue is encoded on a single gene in the fish species studied to date (261, 264, 278–280), in some of which transcript variants have been identified (279, 281). Immunohistochemical analysis revealed that Gata3 is mainly present in lymphocyte-like cells across several teleost tissues, including the gills, spleen, intestine, liver and head kidney of Japanese flounder (278) or Atlantic salmon (282) and can be induced during tissue regeneration, as shown following zebrafish cardiac injury (283). The expression of the *gata3* gene in whole tissues following stimulation or infection corresponds well with expectations for induction in response to extracellular pathogens. Challenge experiments using beta-glucan, LPS, PMA and *V. anguillarum* increase the *gata3* transcript levels in the spleen and/or head kidney of Atlantic salmon and Atlantic cod. In contrast, early after stimulation with the facultative intracellular bacterium *E. tarda* or grass carp reovirus, *gata3* levels are reduced in lymphocytes from the blood, kidney and spleen in Japanese flounder (278) or in the intestine and head kidney of grass carp (*Ctenopharyngodon idellus*) (261), respectively. Whether these opposing responses reflect pathogen-specific signalling, differences in the responding cell populations or distinct kinetics of the immune response remains unresolved.

Reporter-gene analyses revealed that Gata3 from Japanese flounder activates the expression of various immune and non-immune genes (278). In addition, overexpression of *gata3* increases its own promoter activity *in vitro* in salmonid CHSE-214 cells (282) and elevates *il4/13* transcript levels *in vivo* in Atlantic salmon (268), but this does not confer protection against mortality caused by *P. salmonis* and *V. anguillarum* (268). A key unresolved question is whether Gata3 function in teleosts depends on the nature and timing of upstream signals or on cooperation with other lineage-defining factors such as T-bet. Time-resolved, cell-type-specific perturbation of Gata3, combined with analysis of its direct genomic targets and infection outcomes, could test whether Gata3 primarily triggers effector programmes or instead modulates broader transcriptional states during immune activation.

In mammals, several mechanisms have been reported controlling the activity of GATA3 (284, 285). For Japanese flounder, the microRNA miR-4171-x has been identified as a modulator of *gata3* expression during *V.-anguillarum* infection (286). Collectively, these findings highlight miRNAs as an additional regulatory layer that fine-tunes the activity of immune-related transcription factors in fish. As functional validation studies continue to expand, integrating miRNA-mediated regulation with transcription-factor networks will provide a more comprehensive understanding of its evolutionary conservation across vertebrates.

##### 2.3.2.3 Foxp3—the regulator of the regulators

In addition to the above transcription factors that drive effector T_H_-cell differentiation, distinct factors such as FOXP3 govern regulatory pathways, which direct the development of T_reg_ cells.

FOX family transcription factors contribute to the regulation of developmental transitions within T-cell and B-cell states (287). In mammals, FOXO/FOXP factors regulate naïve T-cell and B-cell maintenance, drive the transition from conventional CD4^+^ T-cell programmes towards the T_reg_ lineage and modulate B-cell gene expression and immunoglobulin recombination (287), although their precise mechanistic roles in teleost immunity remain incompletely resolved. Teleost orthologues of *foxp1* and *foxp2* are present in species such as zebrafish and medaka (*Oryzias latipes*), where they promote context-dependent expression in lymphocyte populations and may contribute to lineage-associated transcriptional programmes (288, 289). In grass carp, differential responsiveness of *foxp1b* and *foxp2* during mitogen stimulation of peripheral lymphocytes suggests functional partitioning between lymphoid subsets, potentially reflecting B- versus T-cell-associated programmes (290). The expansion of the *fox*-gene family in some teleosts further indicates lineage-specific diversification of transcriptional control during lymphopoiesis (291).

FOXP3 is a member of the huge forkhead (FOX) family (292) and its activity is thought to depend largely on the C-terminal region, which is well conserved across vertebrates. In contrast, its N-terminal portion shows lower sequence conservation, raising early doubts about functional conservation across vertebrate lineages (293). A broader comparative genomics and structural modelling analysis confirmed that *FOXP3* orthologues are restricted to jawed vertebrates including teleostean fish, but have been lost in avian genomes (293). Experimentally, *foxp3* was initially identified as a single-copy gene expressed in lymphocytes of zebrafish (294) and Nile tilapia (295) but later studies revealed the presence of two *foxp3* paralogues, e.g. in rainbow trout (296).

In zebrafish, only one of the paralogues appears to play a relevant role in immune regulation, since *foxp3a* is expressed by Cd4^+^ cells with typical lymphoid morphology, but also by stromal cells of the head kidney and other non-lymphoid hematopoietic populations (297). Notably, *foxp3* expression has additionally been detected in epithelial-like cells of the stomach mucosa in Nile tilapia (295). Immune stimulation supports a conserved inducibility of *foxp3* in fish: exposure to LPS and/or PHA elevates *foxp3* expression in the spleen of zebrafish, rainbow trout and Nile tilapia (202, 296, 298). In line with these findings, LPS stimulation increases *foxp3* levels in salmon head kidney cells (299), whilst injection with *A. salmonicida* induces *foxp3* expression in the gills, intestine and spleen of Nile tilapia (298).

Functional studies provide evidence that Foxp3 contributes to the control of T-cell activation in teleosts, although the extent to which it fulfils a canonical T_reg_ function remains to be established. Heterologous expression of zebrafish *foxp3* in HEK-293 cells revealed that Foxp3 can homodimerise and interact with NF-κB/Rel and Nfat, thereby reducing their transcriptional activity (294). Consistent with an inhibitory role, *foxp3* overexpression in developing zebrafish embryos increases *foxp3* expression whilst reducing *il17* expression (294). More compelling evidence comes from genetic disruption: zebrafish *foxp3* mutants exhibit reduced survival, growth retardation and immune dysregulation, including altered lymphocyte-to-myeloid ratios, excessive T-cell accumulation, splenomegaly and chronic peripheral inflammation accompanied by increased expression of pro-inflammatory cytokines and T-cell proliferation (297, 300, 301). Together, these findings indicate that Foxp3 restrains lymphocyte activation and inflammatory responses in fish, but whether this reflects direct repression of inflammatory transcriptional programmes, impaired T_reg_ development or both is not yet clear.

Additional evidence suggests that Foxp3 expression is dynamically regulated during immune activation. In rainbow trout, the co-culture of splenocytes stimulated with Ifng and *Piscirickettsia salmonis* leads to a pronounced up-regulation of *foxp3* expression compared with *gata3* or *tbx21*, suggesting that interactions between antigen-presenting-like cells and Cd4^+^ lymphocytes promote polarisation towards a T_reg_ phenotype (302). The expression of *foxp3* is regulated by the mTOR signalling pathway (303). Accordingly, treatment with the macrocyclic lactone rapamycin increases Foxp3 protein levels in the thymus, head kidney, spleen and gills of grass carp, indicating conserved mTOR-dependent regulation in teleosts (304). However, these observations do not indicate that mTOR directly controls Foxp3 transcriptional activity or that altered Foxp3 abundance is secondary to broader changes in lymphocyte activation or metabolism. A key question is therefore how Foxp3 integrates mTOR and NF-κB/Nfat signalling to trigger or maintain regulatory T-cell states in bony fish.

Another member of the FOX gene family is FOXN1. This transcription factor is expressed primarily in thymic epithelial cells rather than in T cells and plays a crucial role in establishing the thymic microenvironment required for the recruitment and differentiation of T-cell progenitors (305). In contrast to mammals, zebrafish carrying a deleterious *foxn1* mutation retain substantial thymic lymphopoietic activity, indicating that T-cell development in teleosts is only partially dependent on Foxn1 function (306). These findings suggest that early vertebrates possessed a robust and redundant regulatory network controlling thymopoiesis. During mammalian evolution, however, this network appears to have been remodelled, resulting in a thymic developmental programme that is predominantly dependent on FOXN1.

#### 2.3.3 Orchestrators of B-cell identity and differentiation

B cells employ a transcriptional programme distinct from that of T cells. Comparative analyses increasingly support the existence of a deeply conserved regulatory architecture (307, 308), now reinforced by single-cell RNA sequencing (scRNA-seq) studies that resolve high-resolution hematopoietic landscapes and reveal broadly homologous B-cell trajectories across vertebrate evolution (309–313). These data indicate that a core transcriptional module comprising *rag1, rag2, tcf3, ikzf1, ebf1, pax5, prdm1* and *xbp1* underpins B-cell specification and differentiation (**Figure 4**), suggesting that these key regulatory mechanisms evolved before the divergence of teleosts and tetrapods (313).

**Figure 4:**
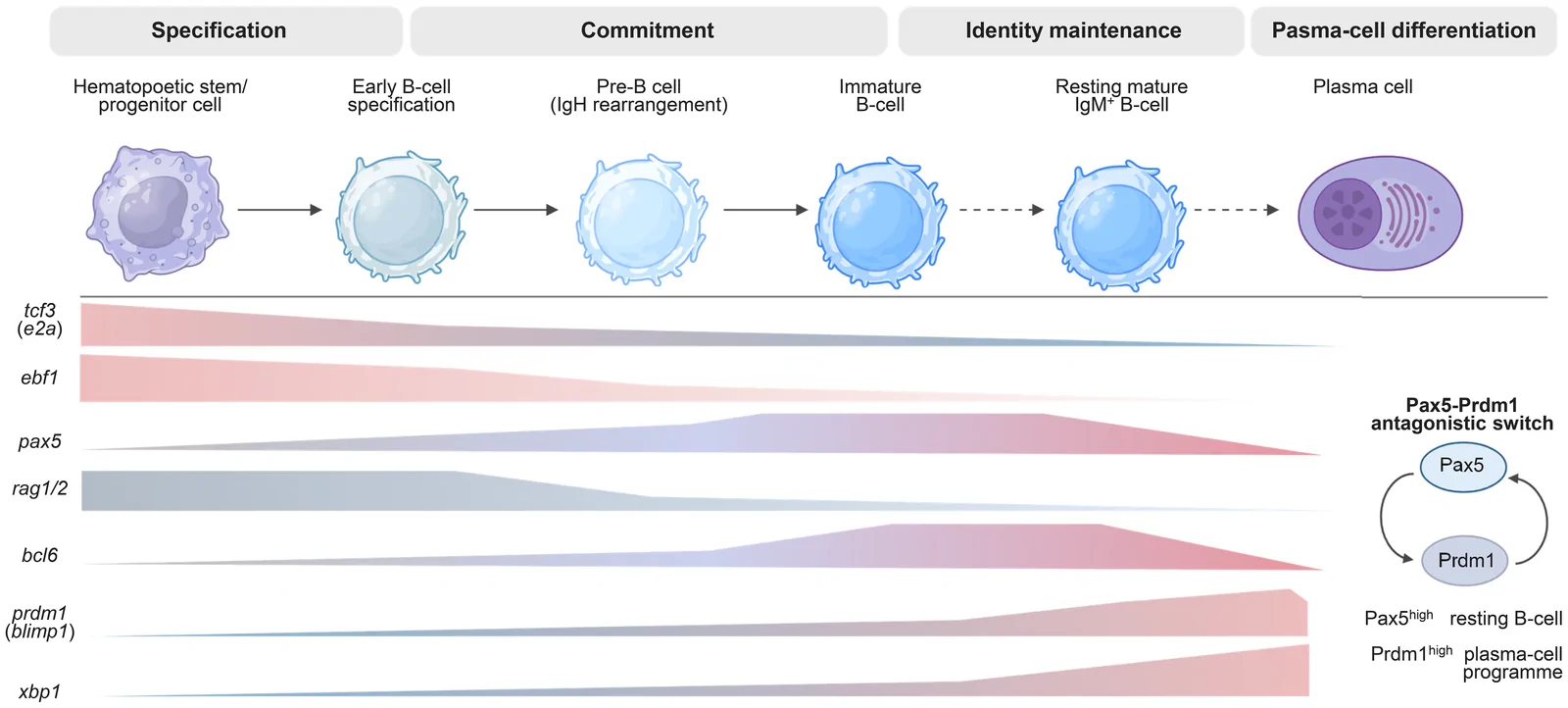
Transcriptional regulation of B-cell identity and differentiation in teleosts. Schematic overview of the conserved transcription-factor network governing teleost B-cell development from early specification to antibody-secreting plasma cells. The Tcf3–Ebf1 axis promotes B-lineage specification and immunoglobulin recombination, whereas Pax5 establishes and maintains B-cell identity. During terminal differentiation, the Pax5–Prdm1/Bcl6 regulatory switch promotes plasma-cell formation, followed by Xbp1-mediated secretory adaptation and immunoglobulin production. The core regulatory module is broadly conserved across vertebrates, although the transcriptional mechanisms underlying teleost germinal center-like responses and affinity maturation remain incompletely defined. Created with BioRender.com.

B-lineage specification is driven by the coordinated activity of the E-protein and EBF1 regulatory axis, which establishes B-cell identity through chromatin remodelling and activation of immunoglobulin-associated gene networks (314, 315). In mammals, E2A (encoded by *TCF3*) is essential for early B-cell development and promotes the expression of recombination-activating genes *RAG1* and *RAG2* and immunoglobulin-locus accessibility (316–318). This enhancer-binding function is broadly conserved in teleosts, where Tcf3-related factors directly regulate immunoglobulin enhancer activity (319–321). Teleost genomes exhibit functional diversification of this module, with transcription factor 12 (Tcf12)-related factors (*cfeb1/2*) partially compensating for or complementing Tcf3 activity in certain lineages (319, 321, 322). In parallel, early B-cell factor 1 (Ebf1) acts as a central lineage-determining factor whose DNA-binding domain is highly conserved across vertebrates, including zebrafish, cartilaginous fish and mammals, underscoring its ancient role in B-cell specification (323–326). In teleosts such as Nile tilapia, *ebf1* expression is strongly enriched in lymphoid tissues and specifically associated with head kidney B-cell populations, consistent with its role in driving progression from early progenitors towards pre-B cell stages (327).

Commitment to the B-cell lineage is ultimately stabilised by the paired box 5 protein (Pax5), which functions as a master regulator of B-cell identity by reinforcing B-lineage gene expression programmes and actively repressing alternative haematopoietic fates (328). In teleosts such as rainbow trout, *pax5* expression demarcates B-cell populations from early developmental stages through mature IgM^+^ cells (**Figure 4**), and isoform diversity may contribute to regulatory fine-tuning of lineage maintenance (329–334). In addition, *pax5* expression is dynamically regulated during immune challenge, including epigenetic repression via DNA methylation in Nile tilapia, suggesting environmental modulation of lineage stability (334, 335). ScRNA-seq analyses in zebrafish further show that early Pax5^+^ progenitors transiently co-express recombination-associated genes such as *rag1/2* and the DNA nucleotidylexotransferase-encoding gene *dntt*, which are subsequently downregulated as cells mature into resting IgM^+^ B cells, whilst *pax5* expression is maintained to preserve lineage identity (336).

Terminal differentiation into antibody-secreting plasma cells requires repression of the PAX5 programme and activation of a conserved antagonistic switch between BLIMP1 (encoded by *PRDM1*) and BCL6 (337, 338). BLIMP1 is a well-known transcriptional repressor that regulates diverse innate and adaptive immune processes through the suppression of lineage-specific transcriptional programmes, including the B-cell and T-cell identity programme and NK-cell regulatory networks (329, 339). The structure of the *prdm1* gene and its promoter sequence have already been analysed in Japanese flounder (340, 341). In teleosts, functional studies in medaka demonstrate that *prdm1* paralogues (*prdm1a* and *prdm1c*) directly repress *bcl6a*, indicating that the cis-regulatory architecture of this plasma cell differentiation axis is evolutionarily ancient (342). As Blimp1 activity increases, it enforces plasma-cell fate through chromatin remodelling, including repression of Pax5 target genes (343). This results in a reciprocal distribution of Pax5-high resting B cells and Blimp1-high antibody-secreting cells (**Figure 4**), particularly in mucosal immune tissues such as the gills and intestine, reflecting specialised compartmentalisation of teleost humoral immunity (334, 343). Concurrently, downstream components of the early B-cell programme, including *tcf3* paralogues, undergo epigenetic remodelling to support activated B-cell expansion during this transition (320).

Following terminal differentiation, *xbp1* (coding for X-box binding protein 1) is upregulated to support the secretory adaptation required for high-rate immunoglobulin production. Beyond its role as a plasma-cell marker in teleost immune systems (309, 312, 313, 344), Xbp1 functions as a central effector of the unfolded protein response, coupling ER stress to transcriptional programmes that expand secretory capacity. In zebrafish, Ire1-mediated unconventional splicing of *xbp1* mRNA generates the active transcription factor Xbp1s, which drives expression of genes required for protein folding, secretion and ER biogenesis, whilst the unspliced isoform participates in transcript regulation under basal conditions (345, 346). This mechanism links terminal B-cell differentiation directly to cellular stress adaptation, ensuring survival and functionality of high-output antibody-secreting plasma cells.

Although teleost fish are capable of antigen-driven B-cell responses involving somatic hypermutation and clonal selection in germinal centre-like microenvironments (347), the transcriptional networks underlying these processes remain incompletely defined. In mammals, germinal centre reactions are governed by a coordinated regulatory programme involving BCL6, BACH2, IRF4 and MYC, yet whether teleosts employ a homologous transcriptional module or an alternative regulatory architecture remains unresolved, representing a major gap in understanding the evolution of affinity maturation (307). The development of transgenic IgM-reporter zebrafish lines, combined with single-cell transcriptomic mapping, has nevertheless demonstrated that temporally dynamic B-cell developmental programmes and larval-to-adult transitions in lymphopoiesis are conserved features of jawed vertebrate immunity, coinciding with the emergence of a fully functional adaptive immune system (348).

Overall, B-cell development in teleosts is governed by a deeply conserved transcription-factor network that mirrors mammals, spanning lineage commitment, identity maintenance and plasma cell differentiation, though key aspects remain still unresolved.

#### 2.3.4 Transcriptional immune programmes in fish

The molecular interactions described in the sections above illustrate a recurring theme in immune evolution: many key regulators of innate immunity are derived from ancient protein families. Members of the NF-κB, IRF and STAT families represent prominent examples of such conserved transcriptional regulators, which have been adapted throughout metazoan evolution to control diverse aspects of host defence (326). Because of this evolutionary conservation, patterns of transcription-factor usage in mammals provided valuable insights into analogous immune mechanisms in fish. By studying these conserved transcriptional programmes, we can better understand how fundamental principles of immune defence have been maintained and adapted across vertebrate evolution, providing a framework for investigating the functional roles of immune cells in teleosts and other non-mammalian vertebrates. Although the molecular transcription-factor machinery is apparently largely conserved across vertebrates, it operates within a different anatomical and cellular architecture.

Teleost immunity differs markedly from that of mammals in its spatial organisation, as bony fish lack the highly organised lymph-node architecture and do not possess the classical germinal-centre organisation observed in mammals (349). Although many transcriptional regulators involved in lymphocyte differentiation, activation and antibody responses are conserved, the cellular and anatomical contexts in which these factors operate differ substantially. The distinctive, unique nature of certain teleost immune cell types, namely nucleated erythrocytes (350), phagocytic B cells (351) and IgT^+^ B cells associated with mucosal immunity (6), provides a further reason why transcription-factor regulation cannot simply be extrapolated from mammalian models. Teleost erythrocytes, for instance, are transcriptionally active and their contribution to immune regulation (352) raises the possibility that transcription factors traditionally associated with leukocyte function may also operate in erythroid cells in teleosts, although the extent and functional specificity of such programmes remain insufficiently characterised.

Similarly, several teleost B-cell populations with phagocytic functions (351) may be governed by transcriptional programmes that combine elements of canonical B-cell differentiation with innate immune functions. Whether such functional plasticity is reflected in distinct combinations of lineage-associated and inflammatory transcription factors remains an important unresolved question. Also, the transcriptional mechanisms establishing and maintaining mucosal B-cell identity, and the extent to which they overlap with or diverge from conventional B-cell programmes, remain incompletely understood. Conserved lineage-associated transcription factors such as Spi1, T-bet, Eomes, Gata3 and Foxp3 provide evidence for evolutionary continuity, but their expression and regulatory interactions may occur in cellular populations and anatomical contexts that have no direct mammalian equivalent.

A particular aspect to consider when analysing immune-relevant transcription factors in teleosts compared with mammals is the occurrence of numerous duplicated genes (**Table 1**) resulting from whole-genome duplications in bony fishes (16, 17). The retained transcription-factor paralogues are not necessarily functionally redundant; the available evidence instead points to a spectrum of evolutionary outcomes, ranging from partial redundancy and compensatory regulation to subfunctionalisation, cell-type-specific expression and stimulus-dependent regulatory divergence (**Table 1**). For example, the two teleost *eomes* paralogues are not co-regulated (273), display distinct expression patterns (270) probably associated with diverged functions, whilst perturbation of one paralogue can alter the expression of another transcriptional regulator (274), suggesting compensatory interactions within the regulatory network. Similarly, duplicated *foxp3* genes show distinct expression patterns across immune-cell populations (297) suggesting functional divergence. These examples illustrate that genome duplication may increase not only the number of transcription factors but also the combinatorial and context-dependent possibilities through which immune signals are translated into transcriptional programmes.

**Table 1:** Published evidence for paralogues of immune transcription factors in teleost fish.

Transcription factor	Teleost paralogues	Species	Sub- functiona- lisation	Cell/tissue specificity	Stimulus-specific regulation	Evidence
Atf1, Atf3, Atf4, Atf6	a, b	*Cyprinus carpio*	unknown	unknown	unknown	Phylogenetic analyses (135)
Atf5, Atf7	n = 4	*Cyprinus carpio*	unknown	unknown	unknown	Phylogenetic analyses (135)
Batf3	a, b	*Cyprinus carpio*	unknown	unknown	unknown	Phylogenetic analyses (135)
Creb1	n = 4	*Cyprinus carpio*	unknown	unknown	unknown	Phylogenetic analyses (135)
Creb5	a, b	*Cyprinus carpio, Danio rerio, Oreochromis niloticus, Takifugu rubripes*	unknown	unknown	unknown	Phylogenetic analyses (135)
Crem	a, b	*Cyprinus carpio, Danio rerio, Oreochromis niloticus*	unknown	unknown	unknown	Phylogenetic analyses (135)
Eomes	1, 2 or a, b	*Danio rerio, Oncorhynchus mykiss, Carassius auratus langsdorfii*	probable	✓	[✓]	Expression profiling, knock-down and overexpression studies (270, 271, 273)
Fos, Fosl1	a, b	*Danio rerio*	unknown	[✓]	✓ (Mapk signalling)	Phylogenetic analyses, expression profiling (134)
Foxp3	a, b	*Danio rerio*, *Oncorhynchus mykiss*	possible	✓ (cell specificity)	✓ (LPS, cytokines, PMA, PHA)	Phylogenetic analyses, expression profiling, *in vitro* stimulation, mutant strains, histopathology (296, 297)
Irf1, Irf2, Irf5, Irf6, Irf7, Irf8, Irf9, Irf10	1, 2	*Oncorhynchus mykiss*	possible	unknown	unknown	Phylogenetic analyses (107)
Irf4	a1, a2, b1, b2	*Oncorhynchus mykiss*	unknown	unknown	unknown	Phylogenetic analyses (107)
Jdp2	a, b	*Danio rerio, Cyprinus carpio, Oreochromis niloticus, Takifugu rubripes*	unknown	unknown	unknown	Phylogenetic analyses (134, 135)
Nfat5	a1, a2, b1, b2	*Salmo salar*	possible	✓	✓ (salinity)	Sequence analysis, expression profiling (98)
Prdm1	a1, a2, b1, b2, c1, c2	*Oncorhynchus mykiss*	likely	✓	✓ (VHSV)	Phylogenetic analyses, expression profiling, *in vitro* stimulation (342)
Stat1	a1, a2, a3, b1, b2	*Oncorhynchus* sp., *Salmo salar*	likely	unknown	unknown	Phylogenetic analyses (198)
Stat3, Stat4	a, b	*Oncorhynchus* sp., *Salmo salar*	unknown	unknown	unknown	Phylogenetic analyses (198)
Stat5	a1, a2, b	*Oncorhynchus* sp., *Salmo salar*	unknown	unknown	unknown	Phylogenetic analyses (198)
Spi1	a, b	*Larimichthys crocea, Plecoglossus altivelis*	likely	✓	✓ (*V. anguillarum*, Ifng)	Expression profiling, blotting, reporter-gene assays, functional knockdown (218, 238)
Tcf3	a, b	*Larimichthys crocea*	unknown	–	✓ (LPS)	Phylogenetic analyses, expression profiling, reporter-gene assays, *in vitro* DNA methylation (320)

✓ = present/reported.

– = absent/not reported

[] = limited evidence

In addition, it is striking that many teleost immune transcription factors apparently remained as single copies or were secondarily pruned back to single-copy status, including teleostean NF-κB/Rel factors, Nfat members 1 to 4, Cebp proteins, Creb3, T-bet, Gata3 or Atf2. Because transcription-factor duplication can disrupt stoichiometric relationships with cofactors, signalling molecules or chromatin modifiers, it may cause inappropriate activation or repression of gene programmes. Purifying selection may therefore act particularly strongly on transcription factors positioned high in regulatory hierarchies and controlling large gene networks, where balanced gene dosage and tightly coordinated interactions are critical (353, 354). Duplicated transcription factors may also evolve divergent regulatory elements, increasing the risk of ectopic expression and potentially interfering with cell-type-specific development and precisely tuned immune responses. Conversely, when duplicated copies retain largely redundant functions, their persistence may provide limited selective benefit, allowing one copy to accumulate deleterious mutations and ultimately undergo non-functionalisation or pseudogenisation (355, 356).

Altogether, teleost-specific genome evolution has generated a panel of duplicated transcription factors that operate within distinct cellular and anatomical contexts, providing opportunities for the rewiring and diversification of transcription-factor networks and ultimately contributing to teleost-specific immune programmes. Nevertheless, direct functional evidence remains sparse for many teleost transcription-factor families, and the relative contributions of redundancy, subfunctionalisation or even neofunctionalisation remain important unresolved questions.

#### 2.3.5 Effective networking: the interaction of transcription factors in teleost fish

Immune-relevant transcription factors rarely act in isolation, but they operate in complex regulatory networks involving positive and negative feedback loops, co-activators, co-repressors and non-coding RNAs, thereby conferring both stability and plasticity to immune responses. Many transcription factors are co-expressed within the same cell, where their relative abundance and interactions contribute to the fine-tuning of gene-expression programmes and immune cell-fate decisions (357).

The literature provides numerous expected, but also surprising insights into the interplay of transcription factors regulating immune-related genes in fish. Examples include the cooperative action of NF-κB and Irf factors in the induction of type-I Ifn genes in Atlantic salmon (358), the regulation of the *il1b* gene by Spi1 and NF-κB (359), as well as the induction of *cxcl8* and *differentially regulated trout protein 1* (*drtp1*) by NF-κB and Cebp factors in rainbow trout (78, 360). The proximal promoter of the gene encoding the soluble Tlr5 (*tlr5s*), which is apparently unique to fishes, contains two AP-1 sites, two Cebp sites, an NF-κB site and a Creb site in the orthologue of Japanese flounder (361). A series of deletion clones derived from the native promoter revealed in HINAE cells that AP-1 and mainly NF-κB enable the basal and flagellin-dependent activation of the *tlr5s* promoter and thus also promote the expression of *tlr5s* itself.

These individual studies can be integrated to form a coherent picture portraying complex processes governed by a combinatorial and hierarchical transcription-factor network. The crosstalk between transcription-factor families as a major principle of vertebrate immunity is, for instance, reflected by the interconnected IRF and NF-κB pathways, which do not operate as independent linear modules but can influence each other at several levels. Irf3 and Irf8 have been shown to modulate Myd88-dependent NF-κB signalling in fish (73), whereas Irf4b and Irf8 can negatively regulate retinoic acid-inducible gene-I like receptor-associated NF-κB activation (362). Another particularly well-characterised example of combinatorial immune regulation is the Ifn-induced Jak–Stat pathway in Atlantic salmon, in which Stat1, Stat2 and Irf9 have been shown to interact physically and to form functional transcriptional complexes (30). In this context, it may be noted that salmonids possess multiple *stat1* genes (**Table 1**), which can modify the composition and potentially the regulatory output of transcription-factor complexes. Although these mechanisms do not necessarily represent direct Irf–NF-κB or Irf–Stat protein–protein interactions, they demonstrate how transcription factors belonging to different regulatory modules can become functionally coupled through shared signalling intermediates. Such regulatory crosstalk may enable immune cells to integrate antiviral and inflammatory signals and to fine-tune the magnitude and duration of the transcriptional response.

A further important consideration is that transcription-factor interactions are highly context-dependent. The same transcription factor may cooperate with different partners depending on cell type, developmental stage, stimulus, chromatin accessibility and the availability of cofactors. Consequently, the functional annotation of individual transcription factors based solely on DNA-binding motifs or transcript abundance provides only a partial description of their regulatory activity. In mammals it has been documented that one transcription factor can function as a coactivator for another: for instance, RELA and IRF3 enhance each other’s activity (363, 364), whereas the glucocorticoid receptor (NR3C1) selectively suppresses RELA/IRF3 complexes at specific promoters (364). NF-κB/Rel factors do not appear merely coincidentally in the previously mentioned examples of interconnected transcription factors. As outlined in Section 2.1.1, they represent central regulatory hubs in immune responses, extending far beyond the activation of innate immunity.

Importantly, NF-κB/Rel factors also play equally pivotal roles in the activation and differentiation of lymphocytes. In the kidney of freshwater carp (*Catla catla*), NF-κB expression increased significantly 24 hours after infection with *Aeromonas hydrophila*. Twenty-four hours later, IgD levels also increased in *A. hydrophila*-stimulated fish, suggesting a link between NF-κB activation and IgD production. Consistently, treatment of primary kidney and spleen-cell cultures with an NF-κB inhibitor reduced the transcript levels of both NF-κB and IgD (365). In Nile tilapia, NF-κB couples TCR and Il17 signals to mediate responses against bacterial *Streptococcus agalactiae* infection. Consequently, the blockade of NF-κB/Rel activity severely impaired T-cell activation and the production of Il17a (366).

Investigations of transcriptional regulation in fish have primarily centred on cis-acting regulatory elements involving expression profiling of individual genes rather than on network-level analyses at the single-cell or chromatin level (78, 359–361, 367). Thus, comprehensive studies of transcription factor co-expression in teleost fish remain limited compared with those in mammals. However, the systematic integration of complementary approaches would be particularly powerful for the reconstruction of cell-type- and stimulus-specific transcription-factor interaction networks to distinguish conserved regulatory interactions from teleost-specific ‘innovations’ and to determine how duplicated transcription factors contribute to the functional diversification of immune responses. A major step towards understanding transcriptional regulation in fish was the development of the first comprehensive epigenomic atlas of rainbow trout (29). This resource demonstrated that chromatin states and regulatory elements shape gene expression, trait variation and genome evolution more than 100 million years after the salmonid whole-genome duplication. As in mammals, approximately 75% of the rainbow-trout genome is transcriptionally inactive and gene regulation follows conserved vertebrate epigenomic principles, with active promoters and enhancers associated with high gene expression and repressive chromatin marks linked to gene silencing (29).

The ENCODE project systematically identified functional elements across vertebrate genomes (368), integrating various assay types (see Section 2.4) to provide a multi-layered annotation of genome function beyond protein-coding genes. Human gene regulatory networks encompass well over 100,000 interactions (369), which vary depending on the physiological state of the individual (370). Although only a fraction of these interactions is understood in humans, knowledge of gene regulatory networks in bony fish remains far more limited. Characterising transcription-factor interactions in teleosts, including validating interactions predicted from high-throughput genomics data, will require substantial time and resources. Consequently, a comprehensive ‘Teleost Gene Regulatory Network Project’ is unlikely to be feasible in the near future; the scale of effort required for such ambitious initiatives has been highlighted convincingly in an insightful Perspective article (370). Nevertheless, the experimental validation of hypothetical interactions between transcription factors and their downstream targets should be pursued on a much broader scale and within systematically coordinated, stepwise studies that enable the direct identification of causal molecular mechanisms.

Insights into promoter activity are advancing our understanding of fish biology across diverse physiological and evolutionary processes. For example, CpG density in gene promoters can predict the lifespan of fish, with negatively weighted promoters enriched for genes involved in B- and T-cell receptor signalling and NF-κB/Rel activation, suggesting these pathways may promote longevity (371). Fish models such as medaka have also been used to investigate the relationship between epigenetic modifications, nucleosome positioning and DNA sequence using supervised machine learning (372). Beyond basic biology, transcription factors are central to translational research because they regulate immune checkpoint genes exploited by tumours to evade host immunity, making them key targets for checkpoint-inhibitor immunotherapies (373). Comparative studies of conserved transcription factors and their regulatory networks in teleosts can advance this field, especially in the case of experimentally tractable models enabling *in vivo* analyses of transcriptional regulation, immune cell development and host–pathogen interactions.

### 2.4 Charting transcriptional tides: techniques for investigating teleost transcription-factor functions

Over the past decades, a wide array of techniques has been developed to probe transcription-factor identity and function at multiple levels, leading to an exponential growth in our understanding of gene regulation and the transcriptional outcomes of transcription factor-mediated signalling. Importantly, sequence analysis and expression profiling, on the one hand, and molecular evidence of transcriptional activity and functional perturbation, on the other hand, provide different levels of support and should not be interpreted equivalently. Nowadays, traditional approaches such as phylogenetic analyses and reporter-gene assays are increasingly complemented by transcriptomics, machine-learning-based bioinformatics and knockout studies (**Figure 5**), providing a comprehensive view of global and cell-specific transcription-factor networks. Together, these methods extend decades of valuable work on individual regulators in model systems to a systems-level understanding of gene regulation.

**Figure 5:**
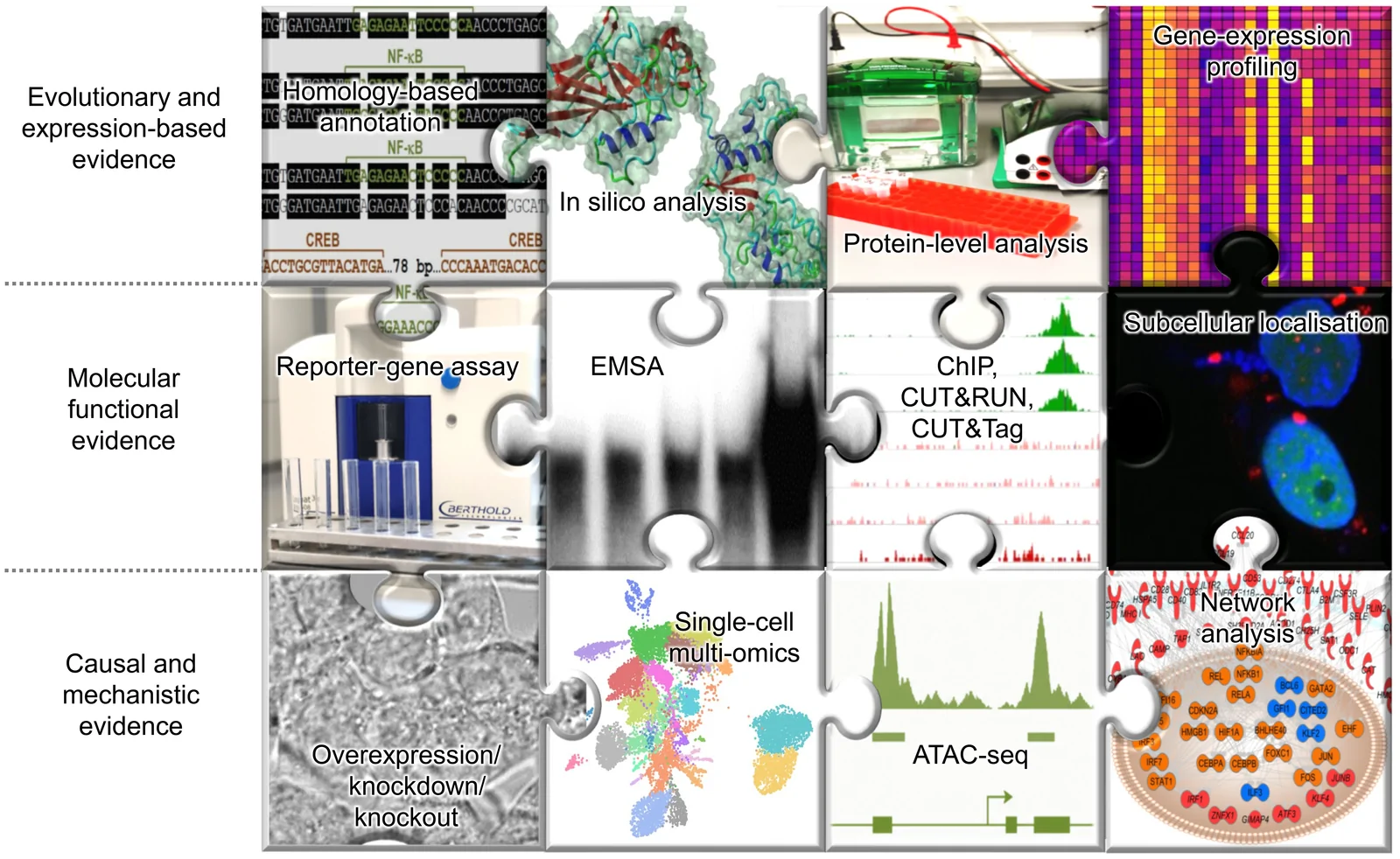
Major experimental approaches for evaluating transcription-factor function in teleost immunity. The approaches are grouped into three levels of evidence according to the type of biological information they provide. Evolutionary and expression-based evidence establishes transcription-factor identity, evolutionary conservation and patterns of expression. Molecular functional evidence provides evidence for transcription-factor activity, DNA binding and transcriptional regulation. Causal and mechanistic evidence tests whether a transcription factor is required or sufficient for a defined transcriptional or cellular response through genetic or functional perturbation, integrating transcriptional, chromatin and network-level information to resolve how transcription factors regulate complex immune programmes. The evidence levels are complementary rather than strictly hierarchical, and the strength of functional inference depends on the combination and experimental context of the approaches applied.

#### 2.4.1 Towards identification of teleost transcription-factor orthologues: cloning, gene-expression profiling and blotting approaches

Transcription factors in model organisms, including bony fish, have traditionally been identified using homology- and domain-based computational approaches. Members of the NF-κB/Rel and Irf families were amongst the first immune-relevant transcription factors characterised in teleosts in the early 2000s (374, 375). These pioneering studies primarily focused on identifying teleost orthologues and characterising their structural features, relying on BLAST-based sequence similarity searches (376), occasionally ‘creative’ targeted primer design, molecular cloning, sequence comparisons and phylogenetic analyses (377).

This methodological framework was subsequently applied to the identification and characterisation of numerous additional immune-related transcription factors in bony fish (378). Despite major advances in genome sequencing, homology-based annotation remains the foundation for transcription-factor identification and typically combines sequence-similarity searches against well-annotated reference genomes using orthology inference tools such as OrthoFinder (379), whilst domain-based classification relies on the detection of conserved DNA-binding domains using resources such as SMART, Pfam, InterPro or HMMER (380–382). These approaches are often complemented by motif-based analyses using curated databases such as Genomatix/MatInspector (383), JASPAR (384) and CIS-BP (385) to infer DNA-binding specificity and classify the transcription-factor families. Although sequence homology and phylogenetic relationships support assignment to a particular transcription-factor family and may indicate conservation of molecular function, they do not inform about the specific role in immune regulation.

Functional association can be inferred from constitutive tissue- or cell-type-specific expression and changes in transcript abundance following pathogen exposure, cytokine treatment or other immune stimulation. Altered expression alone, however, does not demonstrate transcriptional activity, DNA binding or direct regulation of downstream genes. Early expression studies were, in part, based on Northern blotting (67), which at that time represented the most reliable method for quantifying specific transcripts under different experimental conditions. This approach was subsequently superseded by real-time quantitative PCR (qPCR) and, later, by multiplex qPCR, which enabled higher-throughput transcript quantification (386, 387). Advances in transcriptomic technologies now enable transcription-factor expression to be profiled at higher cellular and spatial resolution. Bulk RNA-seq (ideally in combination with cell sorting) enables genome-wide quantification of transcription factor-encoding (and, of course, plenty of other) transcripts under different physiological or experimental conditions, whereas sc- and single-nucleus RNA sequencing resolve the gene expression in individual immune cell populations and other relevant cell types (388).

Spatial transcriptomics preserves tissue architecture whilst linking transcription-factor expression to stimulation-dependent and tissue-specific regulatory programmes beyond conventional annotation methods. As outlined above, teleost-specific whole-genome duplications have expanded immune-related transcription-factor repertoires, creating paralogues that must be considered during gene-expression analyses to avoid masking the regulation of functionally distinct gene copies (17, 389, 390).

Since class-I transcription factors are regulated post-translationally, their genes are typically not identified as stimulus-responsive in transcriptomic analyses based on differential mRNA abundance. Instead, their activation is commonly assessed using protein-based approaches such as immunofluorescence microscopy and western blotting (391). These techniques enable the detection of changes in protein abundance, subcellular localisation and—where phospho-specific antibodies are available—the activation status of (immune) transcription factors. In addition, they provide an important complement to transcriptomic analyses by verifying that changes in gene expression are accompanied by corresponding alterations in protein abundance or activation following immune stimulation.

Despite their importance, functional studies of individual transcription factors in teleosts remain relatively scarce, largely because of the limited availability of species-specific immunological assays, including validated antibodies. The development of monoclonal antibodies and their validation is a long and tedious process requiring a diverse range of skills (392). Nevertheless, several research groups have successfully generated species-specific antibodies or peptide antisera against selected teleost transcription factors (321). Others have exploited cross-reactive antibodies raised against mammalian proteins when the corresponding teleost orthologues share high amino acid sequence identity (typically > 90%) with the immunising peptide (99, 304).

#### 2.4.2 Towards functional characterisation of teleost transcription factors: DNA-binding assays and reporter-gene systems

Evidence for direct transcriptional regulation is provided by approaches demonstrating transcription-factor activity or interaction with regulatory DNA, including DNA-binding assays, chromatin occupancy and reporter-gene assays. Such experiments provide stronger support for a direct regulatory function, although their physiological relevance depends on the experimental context and, in the case of reporter-gene assays, on the cellular system used. Early studies relied mainly on heterologous mammalian cell lines, particularly HEK-293(T) (393) or HeLa cells (280), due to their high transfection efficiency. The use of homologous fish cell lines has increased with the availability of readily transfectable systems such as the carp-derived EPC cell line, the salmonid CHSE-214 cell line and the zebrafish ZF4 cell line (394). Most of the functional studies rely on ectopic overexpression of the gene of interest, typically achieved by transfecting cells with an expression plasmid, in which the coding sequence is placed under the control of a strong constitutive promoter. Although transient overexpression often exceeds physiological expression levels and may cause non-specific effects (395), it remains one of the most widely used approaches in fish immunology, largely due to the limited availability of specific antibodies.

Reporter-gene assays remain a central approach for assessing transcription factor-mediated regulation of promoters and enhancers, typically using luciferase-based reporters combined with promoter deletions or targeted mutations to identify functional regulatory elements (396). Such studies have revealed, for instance, that two ISRE sites control the Irf3-dependent induction of *ifn* transcription in crucian carp (397). Linking promoters of lineage-determining transcription factor genes, such as *spi1*, to fluorescent protein reporters enables the *in vivo* analysis of fundamental processes in haematopoiesis and immune system development (31) using confocal microscopy (398). Such reporter constructs serve as excellent markers for tracking cell movements and dynamic transcriptional changes in transgenic embryos and may also facilitate the identification of molecular pathways involved in immune-cell differentiation. In addition, homozygous mutant models, which are still largely restricted to zebrafish, provide a unique opportunity to dissect the primary and long-anticipated roles of individual transcription factors in immune responses. For instance, a study introducing a *foxp3* mutant in zebrafish embryos demonstrated that disruption of Foxp3-mediated regulation of naïve T-cell differentiation into effector T_reg_ cells shifts the T-cell response towards a T_H_1 phenotype, as reflected by increased expression of *tbx21* (300).

EMSAs demonstrate the direct binding of a transcription factor to a specific DNA sequence and, when combined with competition or supershift assays, can confirm the specificity of these protein–DNA interactions. EMSAs have already been used to show that NF-κB from scup (66) and Tcf3 from channel catfish (321) are capable of specifically binding to their corresponding response elements. This method, similar to chromatin immunoprecipitation (ChIP), is considerably less frequently applied in studies of immune-related transcription factors in fish, mainly due to the limited availability of validated antibodies and/or the requirement for high-quality genome assemblies. ChIP, particularly when coupled with high-throughput sequencing (ChIP-seq), enables the identification of transcription factor binding sites across the genome and thereby reveals direct transcriptional targets *in vivo*. In addition, ChIP-seq can be used to map histone modifications using antibodies against specific epigenetic marks, providing genome-wide insights into chromatin states and gene regulatory landscapes. So far, one reported study used ChIP-qPCR combined with luciferase reporter assays and mutagenesis to demonstrate that Stat6, Rel and Irf4 from zebrafish directly bind to the promoters of *irf4a* and *irf4b* (399).

Building on the principles of antibody-guided chromatin profiling established by ChIP-seq, CUT&RUN (Cleavage Under Targets and Release Using Nuclease) and CUT&Tag (Cleavage Under Targets and Tagmentation) have emerged as low-input alternatives that enable more efficient mapping of transcription-factor occupancy and chromatin states. Importantly, these approaches are no longer restricted to mammalian model systems but have already been adapted to teleosts, including zebrafish (400) and Nile tilapia (401). In zebrafish, CUT&RUN was used to identify Pu.1 and Irf8 as decisive factors controlling the expression of *granulin a* during myeloid differentiation, illustrating how genome-wide chromatin profiling can be combined with transcriptomic and functional approaches to identify biologically relevant transcriptional interactions (400).

#### 2.4.3 Towards systems-level analysis of transcriptional regulation in fish: CRISPR/Cas9, ATAC-seq and gene regulatory network inference

Recent technological advances have expanded the toolkit for studying teleost transcription factors, providing evidence for causal function, particularly when these approaches produce reproducible changes in target-gene expression, cellular phenotypes or immune responses. However, altered expression of a downstream gene following perturbation does not by itself demonstrate direct regulation, as the observed effect may be mediated through other transcription factors or signalling pathways.

Functional validation via loss- and gain-of-function approaches interrogate regulatory roles in immune and developmental processes. Knockdown strategies, including siRNA-mediated silencing (218), enable the transient reduction of transcription-factor expression. The functional role of Tcf3 in embryonic medaka was demonstrated using a knockdown strategy involving phosphonic ester–modified peptide nucleic acids (402). Although this method did not demonstrate any immunological function, the methodological approach has been adapted for use with fish and will hopefully soon be applied in an immunological context.

The CRISPR/Cas9 technique enables precise and heritable gene disruption in cell lines and whole organisms (403). For example, *irf3* knockout zebrafish mutants display increased susceptibility to viral haemorrhagic septicaemia and impaired type-I Ifn induction (404), whilst *foxp3a* disruption affects immune regulation during testis development (300). Complementary gain-of-function studies, typically involving plasmid-driven or viral-mediated overexpression, provide a means of assessing the consequences after restoring transcription-factor activity by measuring downstream changes in immune gene expression and cytokine secretion.

In fish models, particularly zebrafish and medaka, these approaches are further supported by *in vivo* tools such as morpholino antisense oligonucleotide-mediated knockdown, which has been extensively applied in early developmental contexts (180). In combination with transgenic reporter lines that enable real-time analysis of transcription factor-dependent gene expression, these models provide important insights into the regulation of immune development and function, including processes in the thymus, spleen and leukocyte populations.

Knocking out genes encoding transcription factors that determine immune cell polarisation, such as *gata3*, *tbx21* or *spi1*, would be expected to impair differentiation into specialised subtypes or promote a shift towards alternative lineages. Similarly, the knockout of immediate-early transcription factors would alter or even abolish innate signalling responses. Since many transcription factors are crucial components of multiple essential signalling cascades, there is a probability that the loss of certain factors might render the cell non-viable. At this point, reference should be made to CRISP-seq technique, which combines pooled CRISPR-based perturbation approaches with scRNA-seq and enables high-resolution mapping of gene regulatory networks and provides a powerful framework to functionally validate transcription-factor activity and downstream effects (405).

The use of CRISPR/Cas9 as a molecular engineering tool emerged around the same time as assay for transposase-accessible chromatin using sequencing (ATAC-seq) (406). This genome-wide method for identifying regions of accessible chromatin exploits the preferential insertion of a hyperactive Tn5 transposase into nucleosome-free DNA; the resulting accessibility profiles allow the identification of active regulatory elements and the inference of transcription-factor activity through motif enrichment and footprinting analyses. The first study addressing the regulatory basis of the teleost Ifn system combined ATAC-seq, ChIP-seq (H3K27ac/H3K27me3) and RNA-seq following poly(I:C) stimulation. Through motif-enrichment analysis in accessible chromatin regions, this study identified putative transcription factors involved in the antiviral response of Atlantic salmon: Irf8, Irf9, Stat1 and Stat2 constitute a conserved core of Ifn-dependent gene regulation, whereas Stat6, Prdm1, Irf6 or Bcl6 represent additional candidate regulators (30). It is important to emphasise that such predictions remain largely computational and require experimental validation, since regulatory network models generally rely on indirect measures such as co-expression or differential expression.

Recent advances in deep learning are poised to transform transcription-factor research by enabling the inference of complex regulatory relationships from large-scale multi-omics datasets. In particular, the integration of RNA-seq, ChIP-seq, ATAC-seq and comparative genomic data is expected to improve the prediction of transcription-factor binding, target-gene identification and gene-regulatory network reconstruction, whilst reducing the need for exhaustive experimental screening. These regulatory network inference approaches are especially valuable in non-model organisms, including teleost fish, to shift the field from largely descriptive and hypothesis-driven analyses towards predictive and experimentally testable models of immune-gene regulation across vertebrate lineages.

## 3 Conclusions and perspectives

Research into immune-relevant transcription factors in teleost fish links molecular mechanisms to the immune functions of aquatic organisms, informs disease-intervention strategies in aquaculture and expands our understanding of immune-system evolution. Although transcription-factor interactions may be evolutionarily conserved across vertebrates, they are often dynamic and context-dependent and remain particularly poorly characterised in teleost fish. Even orthologues of mammalian transcription factors in fish remain unidentified, whilst the functions of most teleost transcription factors have yet to be elucidated. More broadly, the complexity of transcription-factor networks and their interactions is not yet fully understood, even in the relatively few mammalian models studied, leaving numerous hypotheses, only a limited number of which have been experimentally validated. Moreover, whilst the activation of immunity has been studied extensively, far less is known about how environmental stressors repress immune-cell transcriptional programmes, especially in farmed fish.

Environmental and physiological factors represent an important and still insufficiently explored dimension of teleost transcription-factor networks. Immune transcription is not regulated in isolation but is embedded within physiological responses to nutritional status, temperature, salinity, hypoxia, photoperiod and stressful conditions. These factors can modulate signalling pathways and transcription-factor activity, thereby linking environmental perception and physiological state to immune-gene regulation. Such interactions may be particularly important in aquatic ectothermic teleosts, in which environmental conditions directly influence metabolism, endocrine regulation and immune function. Rather than acting as independent pathways, environmental and immune signals are therefore likely to converge on partially overlapping transcription-factor modules, potentially altering the magnitude, duration and cellular specificity of immune responses.

Little is known, for instance, about how diurnal dynamics influence transcription factor activity in fish. In salmon aquaculture, such temporal regulation is fundamentally disrupted when smoltification is induced by continuous light exposure (407), which can compromise immune function and disease resistance (408). Given that immune-relevant transcription factors in fish can be highly sensitive to light stimuli (145), understanding the transcriptional networks that govern immune responses during the critical window of smoltification and subsequent seawater transfer could reveal whether (even modest) adjustments to salmon-rearing protocols might mitigate immune suppression. Beyond these more specific research questions, transcription-factor networks constitute informative molecular signatures that are likely to reflect key physiological states. Transcriptional programmes elicited by distinct pathogens provide valuable insights into host–pathogen interactions and reveal conserved or disease-specific immune mechanisms. This may be particularly relevant in the context of emerging diseases in aquaculture (409, 410) and for evaluating immunoprophylactic strategies such as vaccination (411).

Against this background, several specific research priorities emerge. First, the functional consequences of whole-genome duplication for immune transcription-factor networks require systematic investigation. Comparative perturbation of retained paralogues, combined with transcriptomic and chromatin-level analyses, should determine whether they regulate overlapping or distinct regulons and whether their functions are cell-type- or stimulus-specific. Second, cell-type-specific regulatory landscapes remain poorly defined. Integrating single-cell transcriptomics with chromatin-accessibility profiling should reveal which regulatory elements and transcription-factor programmes operate in defined immune-cell populations, including teleost-specific or functionally divergent populations, such as nucleated erythrocytes and mucosal or phagocytic B-cell populations. Such analyses could distinguish broadly expressed transcription factors from those that acquire cell-type-specific regulatory functions. Third, predicted transcription-factor interactions and target genes require direct experimental validation. Chromatin-based approaches, DNA-binding assays and reporter-gene systems should determine whether transcription factors, such as those belonging to class I or class II, occupy shared regulatory elements and cooperate or antagonise one another. Fourth, compensatory regulatory circuits remain largely unresolved. Single- and combined-paralogue perturbations could reveal whether duplicated transcription factors buffer one another and whether such compensation depends on cell type, immune stimulus or environmental conditions. Fifth, single-cell multi-omics should increasingly be coupled with genetic perturbation. Integrating cell-resolved transcriptomic and chromatin profiles with CRISPR/Cas-based perturbation would enable causal testing of predicted regulatory relationships and reveal how transcription-factor networks respond to immune and environmental challenges.

These research priorities should be applied broadly across immune-relevant transcription factors to establish a more comprehensive and mechanistic understanding of teleost transcriptional regulation (**Figure 5**). At the same time, they may help address the pronounced imbalance in the current evidence base: whilst some transcription factors, such as Stats, have been studied in considerable detail, others, including NF-κB/Rel and AP-1 factors or the fish-specific factors Cebp1 and Spid, remain only superficially characterised, whilst certain factors, such as Cebpz, have yet to be functionally defined. Applying the above approaches across both well-characterised and understudied transcription factors will help determine which regulatory principles are broadly conserved and which reflect teleost-specific diversification. Future progress will therefore depend not simply on expanding the number of transcription factors investigated, but on exploring causal, cell-resolved and evolutionarily informed regulatory models.

Taken together, the wide range of methodologies established in recent years is poised to transform modern fish immunology by providing mechanistic insight into transcriptional immune programmes. Applying these approaches will advance our understanding of how complex immune responses are coordinated, help identify transcriptional markers and dysregulated pathways associated with disease and uncover the molecular redundancies and intercellular cross-talk that ensure the robustness of immune defence.
